# Wnt-activated osteocyte-driven 3D-bioprinted scaffolds orchestrate bone remodeling, angiogenesis and innervation to accelerate critical bone defect repair

**DOI:** 10.1093/rb/rbag124

**Published:** 2026-06-16

**Authors:** Yangxi Liu, Jian Huang, Shan Li, Xiaolin Tu

**Affiliations:** Laboratory of Skeletal Development and Regeneration, College of Laboratory Medicine, Chongqing Medical University, Key Laboratory of Clinical Laboratory Diagnostics (Chinese Ministry of Education), Chongqing 400016, China; China Western Institute of Digital Intelligent Medicine, Chongqing Medical University, Chongqing 401329, China; Laboratory of Skeletal Development and Regeneration, College of Laboratory Medicine, Chongqing Medical University, Key Laboratory of Clinical Laboratory Diagnostics (Chinese Ministry of Education), Chongqing 400016, China; China Western Institute of Digital Intelligent Medicine, Chongqing Medical University, Chongqing 401329, China; Laboratory of Skeletal Development and Regeneration, College of Laboratory Medicine, Chongqing Medical University, Key Laboratory of Clinical Laboratory Diagnostics (Chinese Ministry of Education), Chongqing 400016, China; China Western Institute of Digital Intelligent Medicine, Chongqing Medical University, Chongqing 401329, China; Laboratory of Skeletal Development and Regeneration, College of Laboratory Medicine, Chongqing Medical University, Key Laboratory of Clinical Laboratory Diagnostics (Chinese Ministry of Education), Chongqing 400016, China; China Western Institute of Digital Intelligent Medicine, Chongqing Medical University, Chongqing 401329, China

**Keywords:** Wnt-activated osteocytes, osteoclastogenesis, neurovascularization, 3D bioprinting

## Abstract

Effective repair of critical bone defects necessitates biomaterials that simultaneously couple bone remodeling (osteogenesis-osteoclastogenesis) with neurovascularization. However, existing multifunctional systems face bottlenecks, including co-culture instability and unpredictable release, due to their reliance on multiple cell types or growth factors. To address this, we developed a 3D-bioprinted scaffold by co-encapsulating SKL2001 (S24)-treated osteocyte osteogenic microenvironment (SOOME) and BMSCs in a gelatin methacryloyl (GelMA) hydrogel within a polycaprolactone (PCL) framework, which is referred to as the SOOME/BMSC@PCL scaffold. In a murine critical-sized calvarial defect model, the SOOME achieved 52.3% bone area fill at 8 weeks, a 5.1-fold increase over the BMSC-only control. It also significantly stimulated osteoclast recruitment, accompanied by elevated bone formation, induced abundant H-type vascularization and promoted neural infiltration. Transcriptomic analysis identified *Vgf* as a differentially expressed gene that partially contributed to the multifunctional integration of SOOME. This research establishes a simpler and more controllable strategy for synergistic regulation of osteogenic, osteoclastic, vascular and neural functions within a SOOME-centered material system, providing new insights into the functional selection of seed cells and opening avenues for clinical translation of bioactive bone grafts.

## Graphical abstract

**Graphical Abstract:**
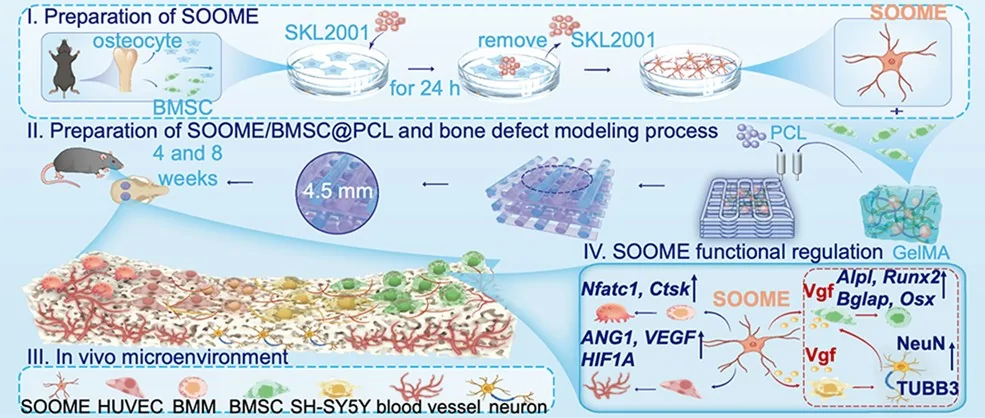

## Introduction

The clinical repair of critical-sized bone defects remains a formidable challenge, with over 20 million surgical interventions following trauma, tumor resection or infection [1]. While autologous bone grafting is considered the “gold standard” with success rates exceeding 90%, it is constrained by limited donor bone supply, secondary trauma and potential complications [2,3]. Clinical orthopedic materials, including metals, ceramics and synthetic polymers, often exhibit inadequate biocompatibility and anatomical fit, with the need for revision surgery in 5–20% of patients within 5–20 years [4,5]. While 3D printing technology has addressed anatomical conformity [6], there remains an urgent need for multifunctional artificial bone grafts capable of integrating the physiological processes of osteogenesis, osteoclastic remodeling, angiogenesis and innervation [7].

Bone tissue engineering (BTE) aims to reconstruct the regenerative microenvironment by integrating biomimetic scaffolds with functional seed cells that serve as a dynamic biological engine, orchestrating matrix mineralization and long-term functional integration. Successful bone regeneration necessitates the coordinated orchestration of osteogenic differentiation, vascular nutrient supply and peripheral neuroregulation [8]. Various cell-based strategies have been reported: Atala et al. first achieved vascularized bone formation using amniotic fluid-derived stem cells (AFSCs) [9]; Pei *et al.* employed Schwann cells to promote pre-vascularization and osteogenesis [10]. Zhou and Zhang *et al.* achieved neurovascular ossification using cell-laden scaffolds containing bone marrow mesenchymal stromal cells (BMSCs), Schwann cells (SCs) or rat aortic endothelial cells (RAECs) [11,12]. Zhang *et al.* tri-cultured human periodontal ligament stem cells (hPDLSCs), human umbilical vein endothelial cells (HUVECs) and neural crest-derived pericytes for osteogenesis, angiogenesis and nerve fibre regeneration [13]. However, these strategies encounter challenges in clinical translation: the most common MSCs undergo senescence or growth arrest after 24–40 passages of *in vitro* expansion [14]; Schwann cells may also secrete angiogenesis inhibitors such as TIMP‑2 [15]; and multicellular systems (three or more cell types) suffer from poor stability of cell coexistence.

More importantly, current strategies neglect the key executor of bone remodeling—osteoclasts. Emerging evidence has confirmed that osteoclasts not only drive bone resorption but also actively contribute to osteogenesis via PDGF-BB and TGF-β signaling [16,17]. To date, no single cell-based platform can simultaneously activate osteogenesis, osteoclastogenesis, angiogenesis and neurogenesis. Therefore, identifying an integrated function carrier capable of reconstructing a physiological bone remodeling microenvironment—including regulation of osteoclastogenesis—is a critical step toward overcoming current bottlenecks in bone tissue engineering.

Osteocytes, constituting approximately 95% of bone cells and being the longest-lived cells in the skeleton, are ideally positioned to coordinate bone metabolism [18,19]. Through their lacunar-canalicular network (LCN), they connect with osteoblasts, osteoclasts, bone marrow cells and the vascular system, enabling them to sense mechanical stimuli and secrete diverse functional factors. Wnt signaling plays a pivotal role in maintaining bone metabolic balance [20,21]. We previously demonstrated that osteocytes, as Wnt target cells, not only directly mediate bone formation [22] and, via TGF-β signaling, promote osteoclastogenesis by upregulating the RANKL/OPG ratio [23], but also promote osteogenic differentiation and angiogenesis *in vitro* [24–26]. These findings highlight osteocytes as promising therapeutic targets for regulating the bone formation-resorption coupling. To harness this potential, we previously used small-molecule Wnt activators in MLO-Y4 cells, thereby establishing an “osteogenic microenvironment” exhibiting both osteogenic and angiogenic properties, preliminarily validating the feasibility of this strategy [24–26]. Nevertheless, as an immortalized cell line, MLO-Y4 struggles to fully replicate the LCN and complex paracrine networks, limiting its utility for investigating deeper mechanisms such as resorption and neural coupling [27].

Given the limitations of MLO-Y4 and the translational bottlenecks of multi-cell co-culture and exogenous growth factor delivery, we developed SOOME—a Wnt-activated primary osteocyte-derived osteogenic microenvironment—and co-encapsulated it with BMSCs in a 3D architecture to recapitulate the native osteocyte-osteoblast unit. In this design, SOOME serves as a paracrine signaling hub but has limited proliferation capacity, whereas BMSCs possess strong osteogenic potential yet depend on microenvironmental cues [28]. Their close co-encapsulation in GelMA facilitates short-range paracrine signaling [29], enabling SOOME-derived factors to guide BMSC osteogenic differentiation. Thus, this design uses only two lineage-related cell types and eliminates the need for external supplementation.

To structurally realize this biological synergy, we employed a PCI3D bioprinting system to fabricate SOOME/BMSC@PCL hybrid scaffolds. The rigid PCL filaments provide mechanical support [30], while the hydrophilic GelMA hydrogel forms an interconnected porous network for nutrient and signal transport [31]. Through endogenous spatiotemporal release of functional factors, SOOME was associated with coordinated osteogenesis, osteoclastic recruitment, angiogenesis and innervation in a murine critical calvarial defect model. Transcriptomic analysis identified the neuropeptide precursor *Vgf* a partial contributor to SOOME-mediated neuro-osteogenic regulation. Collectively, this study establishes a SOOME-centered strategy for multifunctional bone repair, laying a foundation for developing clinically translatable “living” bone grafts.

## Materials and methods

### Materials

#### Experimental animals and ethics statement

Wild-type C57BL/6 mice were obtained from the Chongqing Medical University animal facility. All animal procedures were performed following the ARRIVE guidelines and NIH Publications No. 8023 (revised 1978). All experimental frameworks were pre-approved by the Institutional Animal Care and Use Committee of Chongqing Medical University (Approval No.: IACUC-CQMU-2026-03087).

#### Chemicals and 3D bioprinting materials

Collagenase IA and Alizarin Red S were supplied by Sigma-Aldrich (St. Louis, USA); TRIzol was acquired from Accurate Biology (Hunan, China); and M-CSF and RANKL were sourced from Novoprotein Biotechnology (Suzhou, China). MCE (Shanghai, China) supports the Wnt activator S24. Lyophilized gelatin methacryloyl (GelMA), lithium phenyl-2,4,6-trimethylbenzoylphosphinate (LAP) and polycaprolactone (PCL) were obtained from SunP Biotech (Beijing, China). Matrigel was sourced from Corning Costar (New York, NY, USA).

#### Cell lines and culture reagents

Human umbilical vein endothelial cells (HUVECs) and human neuroblastoma cells (SH-SY5Y) were obtained from ATCC. Gibco (Grand Island, USA) supplied α-Minimum Essential Medium (α-MEM) and Dulbecco’s Modified Eagle Medium (DMEM). Fetal bovine serum (FBS) was purchased from Bioserum (Montevideo, Uruguay). Sigma-Aldrich (St. Louis, USA) provided the β-glycerophosphate, ascorbic acid and dexamethasone. Trypsin digest and penicillin-streptomycin were obtained from Beyotime Biotechnology (Shanghai, China).

#### Biochemical assay kits

The BCIP/NBT alkaline phosphatase (ALP) colorimetric assay, activity assay kit, live/dead cell viability assay, CCK-8 assay and DAPI staining solution were purchased from Beyotime Biotechnology (Shanghai, China). The real-time quantitative PCR kit and the reverse transcription kit were purchased from Accurate Biology (Hunan, China).

### Primary cell culture and functional assays

#### Isolation and culture of primary cells

All primary cells were harvested from 8-week-old C57BL/6 mice. After euthanasia, femurs and tibiae were harvested, and epiphyses were removed. Bone marrow was flushed with α-MEM and centrifuged (800 × g, 5 min). The pellet was used for BMSCs and bone marrow-derived macrophages (BMMs), while the remaining bone shafts were used for osteocytes.

##### Bone marrow mesenchymal stromal cells

The pellet was resuspended in growth medium (89% α-MEM, 10% FBS, 1% penicillin-streptomycin) and cultured at 37°C in a 5% CO_2_ environment with 95% humidity. Medium was changed every 2–3 days, and cells were passaged at 80–90% confluence. Passage 3 (P3) cells were used in all experiments to ensure stable proliferation and differentiation capabilities [32].

##### Bone marrow-derived macrophages

After 24–48 h, the supernatant was removed to collect non-adherent cells. After centrifugation, the cell pellet was resuspended in 10% FBS/α-MEM and 30 ng/mL M-CSF. The medium was replaced every 2 days to yield BMMs, which were used in the osteoclastogenesis assay [24].

##### Osteocytes

Following the method described by Wang *et al.* [24], the bone was cut into small pieces, approximately 1 mm³. In a 37°C water bath, the bone fragments were treated three times with a 1 mg/mL collagenase type I solution at 80 × g for 15 min, sequentially with 4 mM EDTA solution for 5 min and 1 mL of 10% FBS/α-MEM solution for 5 min, for a total of three cycles. Place the treated fragments in 10% α-MEM, seed them into a six-well plate and incubate them at 37°C in a 5% CO_2_ atmosphere with 95% humidity. Primary osteocytes treated with the Wnt activator S24 (60 μM) for 24 h to generate osteogenic microenvironment were designated as SOOME. WTO (wild-type osteocytes) and Ot-free (osteocyte-free) were considered as control groups.

#### ALP staining and activity assay

To assess osteogenic activity [33], cells were washed three times with PBS, fixed with 4% formaldehyde for 5 min. Then, the samples were incubated in the dark at 37°C for 10–30 min using the BCIP/NBT alkaline phosphatase staining kit. The results were recorded with an inverted microscope. For biochemical quantification [34], cells were scraped on ice into 200 μL of PBS (pH 7.4) and then centrifuged at 12 000 × g for 3 min. The supernatant was analyzed using an ALP assay kit, with absorbance measured at 405 nm.

#### Mineralization assay (alizarin red S staining)

To evaluate mineralization [35]: First, cells were pre-cultured in basal medium for 7 days, and then for 14 days in osteogeny in medium containing 0.1 mM dexamethasone, 10 mM sodium β-glycerophosphate and 50 µg/mL vitamin C. Cells were stained with a 0.2% alizarin red S solution for 30 min, observed and recorded under an optical microscope. Next, the samples were placed in a 10% cetylpyridinium chloride solution for 60 min and the absorbance was detected at 562 nm.

#### TRAP staining

To assess osteoclast differentiation [36], cells fixed with 4% formaldehyde were stained by adding 250 μL of the prepared tartrate-resistant acid phosphatase (TRAP) staining solution at 37°C for 10–20 min. Finally, TRAP-positive cells with ≥3 nuclei were quantified and classified as mature osteoclasts under a microscope.

#### Preparation of conditioned medium

Osteocytes were treated with DMSO or S24 for 24 h and then rinsed thrice with PBS to replace the drugs. Then, the cells were transferred to fresh complete medium and cultured for another 24 h. The conditioned medium (CM) was carefully pelleted and saved at −80°C. The blank group contained no osteocyte conditioned medium.

#### Tube formation and migration assays

A 24-well plate was pre-coated with 200 μL of Matrigel, and 1 × 10^5^ HUVECs per well were treated with 200 μL of the above CM for 6 h [37]. ImageJ software was used to quantitatively analyze the vascular networks formed by HUVECs.

For the migration assays [38], transwell chambers with polycarbonate membranes (8.0 µm pore) were used. HUVECs or SH-SY5Y cells were serum-starved for 12 h. Then, 800 µL basal medium and 200 µL CM were added to the lower chamber. Approximately 1 × 10^5^ HUVECs or SH-SY5Y were seeded into the upper chamber at 37°C for 48 h to allow them to migrate through the membrane. The lower chamber was stained with crystal violet. Migrating cells were observed and imaged under a microscope, and their numbers were quantified using ImageJ software.

#### SEM of osteocytes

Morphological changes in SOOME were evaluated via scanning electron microscopy (SEM) [39]. Osteocytes treated with S24 (60 μM) for 24 h were seeded onto glass slides in a six-well plate. The cells were treated with pre-chilled 2.5% glutaraldehyde (4°C) for 30–60 min to preserve morphology. After dehydration with ethanol gradients (30%, 50%, 70% and 90%), the samples were transferred to tert-butanol for two 20-min displacements. Finally, the samples were frozen at −20°C and sublimated in a vacuum oven. After vacuum-sputtering a 10–20-nm gold layer, cells were observed under a SEM (SU8010, 5.0 kV).

### 3D bioprinting and characterization of SOOME

#### Fabrication of PCI3D scaffolds

Based on the team’s previous work [40], a dual-nozzle 3D bioprinter was used to fabricate PCI3D scaffolds. One nozzle melted polycaprolactone (PCL) at 95°C and extruded it at 2 mm/s (nozzle diameter: 400 μm; spacing: 1100 μm). The other nozzle extruded GelMA (10% w/v) with 0.5% LAP, containing osteocytes and BMSCs, at 5 mm/s (nozzle diameter: 300 μm; spacing: 500 μm) into the gaps of the PCL framework. The bio-ink was prepared by lyophilizing GelMA foam in α-MEM, then combining it 1:1 with the cell suspension. After precooling at 4°C for 5 min to optimize viscosity. After printing, the structure was cross-linked by blue light irradiation at 405 nm for 10–15 s. Four layers were deposited using a 0°/90° vertical stacking method. The final scaffold was cultured at 37°C and 5% CO_2_, containing three groups: group 1 (Ot free): PCL/GelMA scaffold with BMSCs alone (no osteocytes), group 2 (WTO): PCL/GelMA scaffold with wild type osteocytes (WTO) + BMSCs, group 3 (SOOME): PCL/GelMA scaffold with SOOME + BMSCs.

#### Physicochemical characterization

The surface morphology and interconnected pore structures of the 3D-printed bio-scaffolds were observed via SEM (5 kV, Hitachi, Japan); Surface wettability evaluation: The scaffolds were assessed using the sessile drop method on a contact angle goniometer (OCA15PRO, Germany). 3 μL of deionized water was placed onto different locations of each sample for 5 s, and its contact angle was captured using a high-speed camera and calculated using an ellipse-fitting algorithm. Dynamic Frequency Sweep: Place cross-linked 10% GelMA or cell-laden GelMA on the test platform. Within the linear viscoelastic region, was performed a frequency sweep from 0.158 rad/s to 100 rad/s. To ensure data reliability, the starting frequency was selected to avoid initial instrumental instability. The storage modulus (*G′*), loss modulus (*G″*) and loss factor (tan *δ*) were recorded. The complex viscosity was calculated to evaluate the shear-thinning behavior and the suitability of the bio-ink for the PCI3D process.

#### Cell viability assay

This study used the Live/Dead Viability Assay Kit to quantitatively analyze cell viability in the scaffolds [41]. On Days 1, 4 and 7, the 3D scaffolds were gently washed three times with PBS (pH 7.4) and then stained with 1 μL Calcein AM (1000*g*) and 1 μL propidium iodide (PI, 1000*g*) for 30 min at 37°C in the dark. Fluorescence images were captured. The live/dead cell ratio was then calculated using ImageJ software.

#### Cell proliferation assay

Cell proliferation in the scaffolds was assessed using the CCK-8 assay kits [42] on Days 1, 4 and 7. Briefly, each scaffold was then homogenized, divided into four equal portions and transferred to a 96-well plate. Then, 100 μL of PBS containing 10% CCK-8 reagent was used, and the wells were treated for 2 h in the dark at 37°C and 5% CO_2_. Transfer 100 μL of the supernatant to another blank 96-well plate and measure the absorbance at 450 nm. Blank medium served as the control.

### *Vgf* siRNA knockdown and functional validation

#### siRNA transfection

*Vgf* siRNA-mediated knockdown was performed as described previously [43]. Primary osteocytes at 60–80% confluence were transfected with 1.5 µL of 20 µM *Vgf* siRNA (Wuhan GeneCreate Biological Engineering) with 1.5 µL of Lipofectamine 3000 in 50 µL of OPTI-MEM for 15 min at room temperature. We set up three groups as follows: WTO, SOOME + si-NC (negative control, transfected with disordered non-targeting siRNA) and SOOME + si-Vgf. After 48 h, the cells were harvested for qPCR detection of *Vgf* expression. siRNA sequences are provided in Supplementary Table S4.

#### Effect of Vgf towards BMSCs and SH-SY5Ys

The transfection reagents were removed, and fresh basal medium was used for 1 day. The supernatant was retained and treated with BMSCs for 5 days. Osteogenic differentiation was then assessed. Alternatively, using the above CM to treat SH-SY5Ys, which were examined for neural differentiation and migration ability.

#### Evaluation of neuro-osteogenic feedback

Subsequently, to further explore the interaction between neural signals and bone formation, the effects of SH-SY5Y cells (cultured under different BMSC CM environments) towards BMSC were evaluated using ALP staining, ALP activity assays and qPCR analysis of osteogenic marker genes.

### RNA extraction and gene expression analysis

Total RNA was extracted using TRIzol, and cDNA was synthesized from 1 μg of total RNA using the PrimeScript RT Kit. The cDNA was diluted fivefold and stored at −20°C. qPCR was performed using the SYBR Green Pro Taq HS Premix qPCR Kit. GAPDH was used as a control, and relative mRNA expression levels were calculated using the 2^−ΔΔCt^ method. Values were expressed as mean ± SD from three biological replicates [24]. Primer sequences were listed in Supplementary Tables S2 and S3.

### RNA sequencing analysis

RNA sequencing was performed by Guangke Ande. RNA from six mice was assessed for purity (NanoDrop 2000) and integrity (Agilent 2100). mRNA was enriched with Oligo(dT) beads, fragmented and cDNA was synthesized. Paired-end sequencing (150 bp) was done on Illumina NovaSeq 6000 (∼35.72 GB clean data). Reads were aligned to GRCm39 (release 109) with >97.5% alignment. Differential expression was analyzed using DESeq2 (|fold change| > 2, adjusted *P *< 0.05). Heatmaps were generated with pheatmap in R.

### *In vivo* evaluation of bone regeneration

#### Mouse critical-sized calvarial defect model

We employed a standard mouse cranial defect model (4.5 mm in diameter) to evaluate bone regeneration [44]. Thirty 8-week-old male C57BL/6 mice (22 ± 2.0 g) were selected and randomly assigned to three groups at 4 and 8 weeks: Ot free, WTO and SOOME. The mice received intraperitoneal Avertin (0.2 mL/10 g) for anesthesia and were secured in a stereotaxic apparatus. During surgery, each mouse received bilateral parietal implants of the above group composite 3D scaffolds. After surgery, we sutured the incision with 5‑0 suture. And the animals were allowed to resume *ad libitum* feeding once they had awoken. At 4 and 8 weeks post-implantation, respectively, the animals were humanely killed by CO_2_ asphyxiation, and skull samples were collected and incubated in 4% paraformaldehyde at 4°C overnight.

#### PET-CT analysis

Radiological analysis was performed using a NanoScan PET/CT system (Mediso, USA) with the following parameters: 50 kV, 560 μA and 50 μm resolution [45]. 3D images were reconstructed by PMOD software (PMOD Technologies, Switzerland), with a threshold of 550 [46]. Quantitative analysis of bone regeneration was conducted using 3D-reconstructed images. The area of newly formed bone (BA) and the total area of the defect region (TA) were calculated using ImageJ software, and the percentage of new bone formation was evaluated by the BA/TA ratio.

#### Histological and morphometric analysis

All skull specimens were decalcified in a 14% EDTA solution (w/v in PBS, pH 7.4) for 2 weeks. Then, the specimens underwent dehydration via an ethanol gradient, cleared in xylene and embedded in paraffin. Finally, 5-μm-thick serial sections were prepared using a Leica microtome (Wetzlar, Germany) [47].

Hematoxylin and eosin (H&E) staining was used to examine bone formation zones and osteoblast distribution [48]. After fixation (4% PFA, 24 h) and decalcification (14% EDTA, 2 weeks), samples were dehydrated in graded ethanol, embedded in paraffin and sectioned at 5 μm. Staining was performed using a modified Harris hematoxylin (5 min) and eosin (1 min) protocol, followed by differentiation with acidic ethanol and restaining with ammonia to ensure clear contrast between cell nuclei and cytoplasm [49]. Osteoblast density (N.Ob/T.Ar) was quantitatively assessed with the OsteoMeasure digital video system. Given that osteoblasts were primarily localized in areas of new bone formation rather than on the bone surface, this study selected tissue area (T.Ar) as the reference parameter for quantitative analysis [50].

TRAP staining: Since osteoclasts often fuse to form multinucleated giant cells, making accurate counting of individual cells difficult, the osteoclast surface area (Oc.S) was selected as the evaluation parameter. To objectively assess the effects of different scaffold materials on osteoclast activity, the osteoclast surface area (Oc.S/T.Ar) was standardized for analysis. This standardization method was particularly suitable for experimental models involving continuous new bone formation [51].

#### Evaluation of collagen deposition and osteoblast activity

Picrosirius red staining was used to assess osteoblast activity and examine type I collagen deposition in paraffin sections [52,53]. Decalcified paraffin sections were dewaxed and rehydrated; stained by a 0.1% picrosirius red solution for 10–30 min. Weigert iron hematoxylin was used as a counterstain; sections were washed, dehydrated in graded ethanol, cleared in xylene and mounted in neutral resin [53]. The collagen fiber distribution was observed under an inverted light microscope, and type I collagen (red, coarse fibers) and type III collagen (green, fine fibers) were distinguished in polarized light mode.

#### Analysis of formed blood vessels

To examine H&E-stained sections and assess vascularization in bone tissue. Images were captured under standard lighting conditions using the OsteoMeasure digital video system. The evaluation metric was the number of vessels per mm^2^ of tissue area (N.Vessel/T.Ar). The criteria for vessel identification [54] were: (1) an intact lumen structure, (2) the presence of red blood cells and (3) clearly visible endothelial cell nuclei.

#### IF detection of neurovascular markers

Immunofluorescence (IF) staining was performed to assess the expression of angiogenic (CD31, Endomucin) and neurogenic (β3-tubulin, NeuN) markers *in vivo* [24]. Briefly, paraffin-embedded sections were permeabilized with 0.25% Triton X-100 for 20 min at room temperature, followed by overnight incubation at 4°C with primary antibodies against Endomucin, CD31, β3-tubulin and NeuN (all at 1:200 dilution). After washing, the sections were incubated with fluorescence-conjugated secondary antibodies (1:200 dilution) for 2 h at room temperature. Stained sections were visualized and captured using a confocal laser microscope (Nikon, Sendai, Japan). Quantitative fluorescence intensity analysis was performed using ImageJ software [55]. Three non-overlapping fields of view were randomly selected for each sample, and the average fluorescence intensity of the target region was measured. To eliminate background interference, the background fluorescence intensity of each field of view was subtracted. Normalization was performed using the average fluorescence intensity of the Ot-free group as a baseline (set to 1). The relative fluorescence intensities of the SOOME and WTO groups were expressed as fold changes.

### Statistical analysis

Statistical analysis was performed using GraphPad Prism 9.5.0 software. All quantitative data are presented as mean ± standard deviation (SD) from at least three independent experiments. For comparisons between two groups, Student’s *t*-test was used. For multi-group comparisons, one-way ANOVA followed by Tukey’s *post hoc* test was applied. Two-way ANOVA was used to evaluate the effects of two independent variables and their interaction, followed by Šidák’s multiple comparisons test. For non-normally distributed data, the Kruskal-Wallis test with Dunn’s *post hoc* test was used. A *P* < 0.05 was considered statistically significant.

## Results

### Effect of SOOME on osteogenic differentiation and mineralization of BMSCs

To investigate the effects of SOOME on bone formation, primary osteocytes were isolated from bone tissue fragments of wild-type C57 mice by sequential digestion with type I collagenase and EDTA. We verified osteocytes by assessing the expression of osteocyte-specific markers (*Dmp1, Mepe* and *Sost*) (Figure 1A). Based on previous studies, 60 µM S24—a small-molecule Wnt activator–was selected to activate Wnt signaling in primary osteocytes (designated as SOOME, Figure 1B), as this concentration does not affect cell proliferation and yields optimal activation [56].

**Figure 1:**
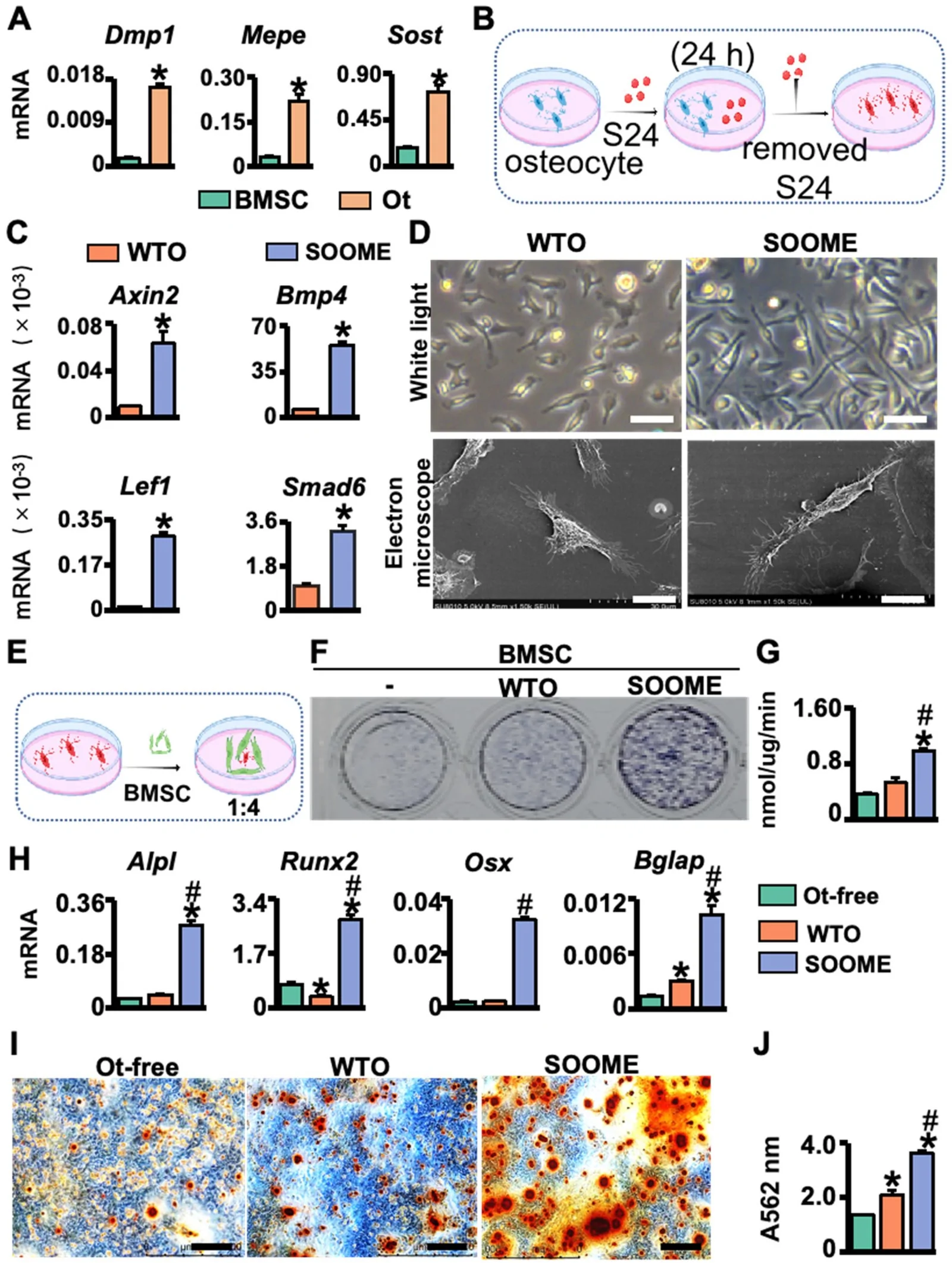
The effect of SOOME on osteogenic differentiation and mineralization of BMSCs. (**A**) qPCR analysis of osteocyte marker gene expression. (**B**) Schematic of SOOME preparation; (**C**) mRNA level of Wnt target genes in SOOME. (**D**) Light micrographs and scanning electron micrographs of morphological changes in SOOME (scale bars: 50 μm for light microscopy, 30 μm for SEM). *P < 0.05 vs. BMSC by *t* test. (**E**) Schematic diagram of SOOME and BMSC (1:4) co-culture. (**F**) ALP staining and (**G**) activity evaluations of BMSCs co-cultured with osteocytes treated with DMSO (WTO) and SOOME for 5 days. (**H**) mRNA level of osteogenic differentiation marker genes detected by qPCR. (**I**) Alizarin Red S staining of BMSCs co-cultured with WTO and SOOME, respectively for 21 days in osteogenic induction medium (scale bar = 50 μm), with mineralization quantification (**J**). Ot-free, BMSC alone, WTO, osteocyte treated with DMSO; SOOME, osteocytes treated with 60 µM S24. *n* = 3 individual experiments. Data were expressed as mean ± SD. *P < 0.05 vs. Ot-free, ^#^*P *< 0.05 vs. WTO by one-way ANOVA.

#### Induction of SOOME

qPCR analysis revealed that SOOME significantly upregulated Wnt target genes (Figure 1C), confirming the enhanced Wnt/β-catenin pathway activity in osteocytes. Light and SEM results displayed that Wnt activation induced morphological changes: DMSO-treated control osteocytes (WTO) exhibited classically triangular cell bodies, whereas SOOME displayed elongated, fibrillar cell bodies and the number of canaliculi pseudopods increased, which may correlate with subsequent functional changes (Figure 1D).

#### Influence of SOOME on osteogenic differentiation and mineralization

To evaluate the effect of SOOME on the osteogenic differentiation of BMSCs, we determined that the optimal co-culture ratio of osteocytes and BMSCs was 1:4, which yielded the best osteogenic differentiation (Figure 1E; Supplementary Figure S1A). Compared to the Ot-free and WTO group, SOOME-treated BMSCs exhibited a significant pro-differentiation effect (Figure 1F and G). ALP staining and quantitative activity tests revealed that ALP activity in the SOOME group was 2.74-fold stronger than that in the Ot-free group and 1.85-fold stronger than that in the WTO group (Figure 1G). Analysis of osteogenic marker gene expression (Figure 1H) revealed that mRNA levels of *Alpl*, *Runx2*, *Osx* and *Bglap* were substantially upregulated in the SOOME group, compared to the Ot-free and WTO groups. Alizarin red S staining and quantitative analysis revealed (Figure 1I and J) that the mineralized nodules formed in the SOOME group were denser, with calcium deposition levels 2.71-fold and 1.73-fold higher than those in the Ot-free and WTO groups, respectively. These data demonstrate that SOOME facilitates BMSCs’ osteogenic differentiation and mineralization.

### Material characterization and biocompatibility assessment of 3D-printed scaffolds

To fabricate the 3D-bioprinted scaffold, we first evaluated the physicochemical properties of the printing materials. As shown in Figure 2A and B, 10% GelMA exhibited excellent extrudability and printing fidelity, and the resulting hydrogel scaffold possessed a highly ordered and interconnected pore structure (Figure 2B-a and b). Enlarged images revealed that cells were uniformly distributed within the hydrogel (Figure 2B-c), and after 24 h of culture, the scaffold’s pore network remained stable with good cell viability (Figure 2B-d–f), confirming that 10% GelMA can provide a stable spatial microenvironment for cells. Viscoelastic analysis revealed that, following photopolymerization, the storage modulus (*G′*) in both the pure GelMA and the cell-laden groups was significantly higher than the loss modulus (*G″*), exhibiting typical solid elastic behavior, indicating that the addition of cells did not interfere with the photopolymerization kinetics of GelMA (Figure 2C). Rheological analysis further confirmed pronounced shear-thinning in both groups; pure GelMA displayed slightly higher initial viscosity due to minor physical network disruption by cell particles, but both groups maintained good extrusion properties and shape retention (Figure 2D).

**Figure 2:**
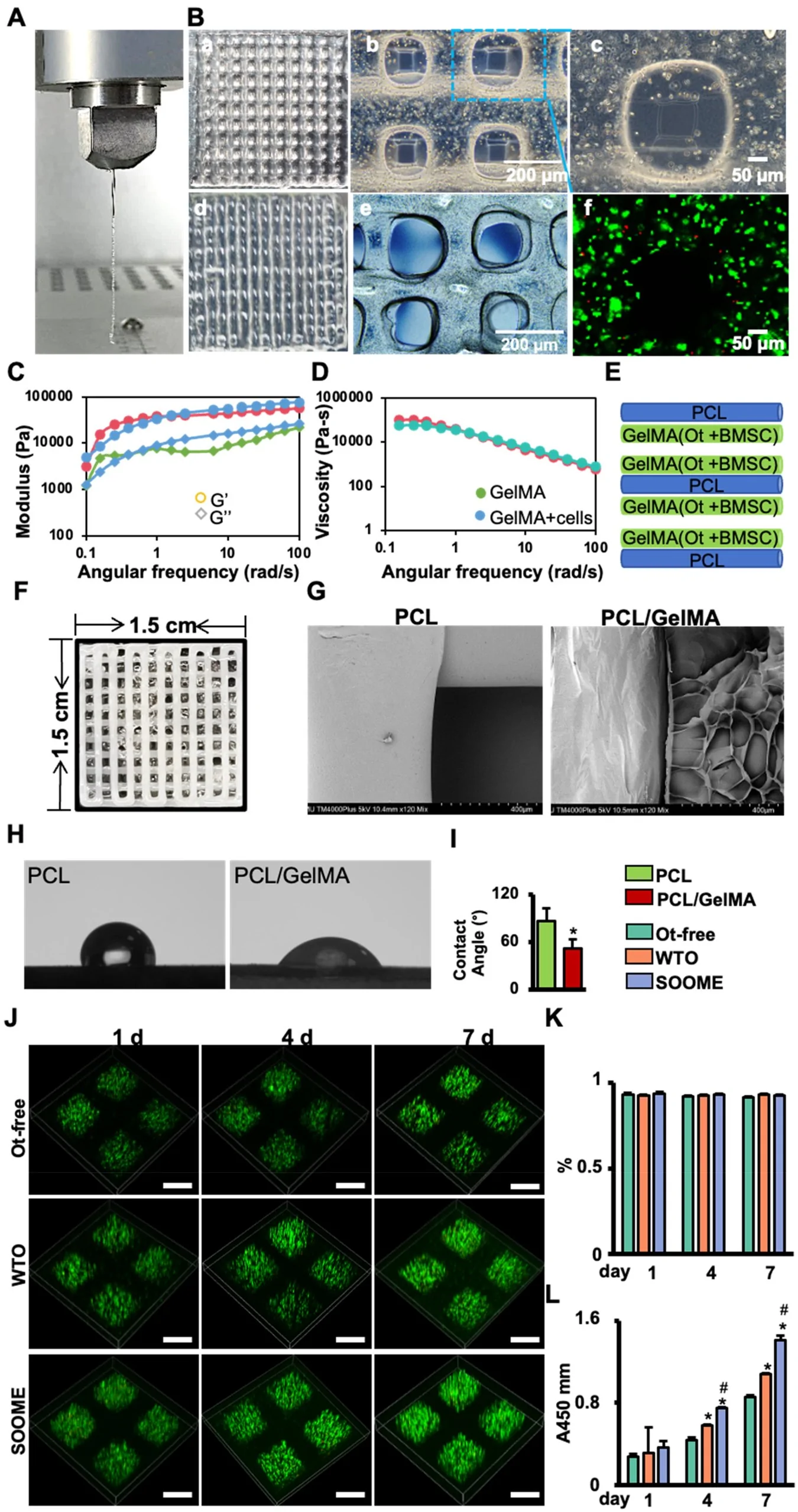
Material characterization and biocompatibility assessment of 3D-Printed scaffolds. (**A**) Representative extrusion image of 10% GelMA. (**B**) Morphological image of 3D printed 10% GelMA hydrogel scaffold: (**a**) gross appearance of the scaffold, (**b, c**) magnified image of the scaffold microstructural details (scale bars = 200 μm and 50 μm, respectively), (**d**) images of hydrogel scaffolds after 24 h of incubation in medium. (**e**) Enlarged image of the scaffold (scale bar = 200 μm), (**f**) live/dead fluorescence staining of the scaffold after 24 h of culture. (**C**) Viscoelastic analysis showing the storage modulus (G′) and loss modulus (G″) of pure GelMA and cell-laden GelMA. (**D**) Rheological characterization illustrating the complex viscosity as a function of angular frequency for both groups. (**E**) Design of the PCI3D scaffold. (**F**) Gross images of a PCI3D scaffold. (**G**) SEM images of the scaffold surface. (**H**) Representative images of water contact angle (WAC) of PCL and PCL + GelMA scaffolds, and quantitative analysis of surface wettability (**I**). (**J**) Live/dead cell assay on days 1, 4 and 7 in the scaffold. Scale bar = 200 μm. (**K**) The live/dead cell ratio and proliferative activity (**L**) were analyzed using ImageJ and the CCK8 assay kit. Ot free: PCL/GelMA scaffold with BMSCs alone; WTO: PCL/GelMA scaffold with WTO + BMSCs; SOOME: PCL/GelMA scaffold with SOOME + BMSCs. Data were expressed as mean ± SD (*n* = 3). *P < 0.05 vs. Ot-free, ^#^*P *< 0.05 vs. WTO, analyzed by two-way ANOVA.

Based on this, we used a PCL-Cell integrated 3D(PCI3D) print system [40] to prepare three types of 3D-printed scaffolds (Figure 2E and F): (1) Ot-free scaffold (containing only BMSCs in GelMA); (2) WTO scaffold (co-loaded with WTO and BMSCs in a 1:4 ratio); (3) SOOME scaffold (co-loaded with SOOME and BMSCs in a 1:4 ratio). SEM observations (Figure 2G) revealed that GelMA formed a highly porous surface microstructure within the PCL framework, facilitating nutrient transport and signal exchange. Water contact angle (WCA) testing (Figure 2H) demonstrated that the integration of GelMA notably improved the hydrophilicity of PCL, reducing the scaffold’s contact angle to 51.6°. This enhanced hydrophilicity facilitates the diffusion of SOOME-derived factors and the recruitment of endogenous cells.

To evaluate cell biocompatibility, PCI3D scaffolds were stained with Calcein AM/PI on days 1, 4 and 7 to assess cell viability. As illustrated in Figure 2J and K, these scaffolds exhibited high cell viability, with live cell rates of 92.9% in the SOOME group, 92.2% in Ot-free scaffolds and 92.7% in WTO groups, respectively. The 7-day CCK-8 assay showed stable linear proliferation across all groups (Figure 2L), with the SOOME exhibiting increased activity compared with the control group at Days 4 and 7. Overall, these results indicate that PCI3D scaffolds effectively support cell survival and expansion, whereas WTO and SOOME promote BMSC proliferation.

### Bone formation by SOOME *in vivo*

#### Establishment of a critical-size parietal bone defect model and implantation of SOOME

A model of critical-size parietal bone defects (4.5 mm) in C57BL/6 mice was constructed (Figure 3A) [57]. SOOME and the control groups were processed into circular scaffolds (Supplementary Figure S2A) and implanted into the defect site (Supplementary FigureS2A-a). At 4 and 8 weeks post-implantation, we examined the effects of bone repair. Macroscopic assessment of the scaffold was performed at 4 weeks (not shown) and 8 weeks (Supplementary Figure S2A-b).

**Figure 3:**
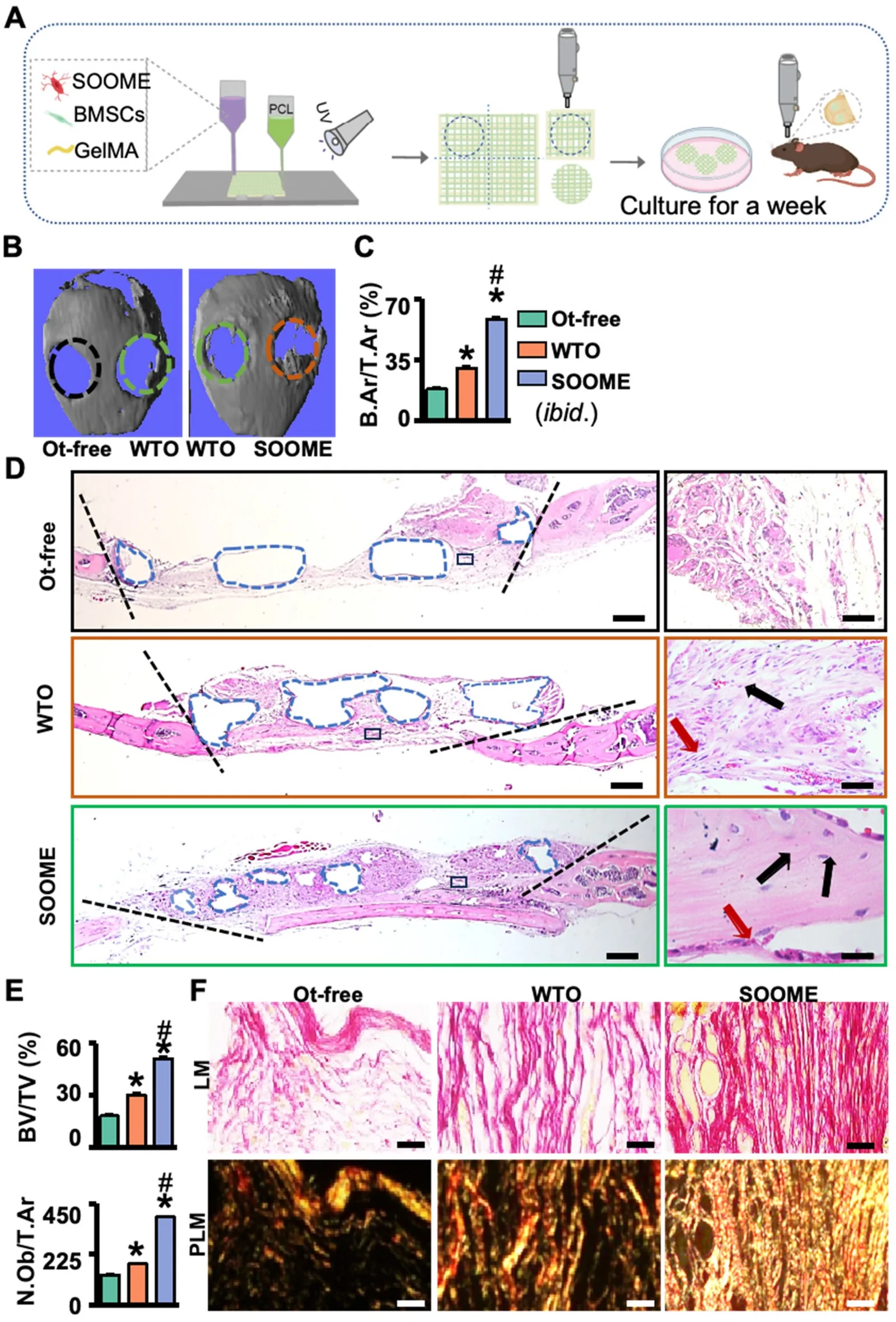
Bone formation by SOOME *in vivo*. (**A**) Schematic of critical-sized calvarial defect model establishment. (**B**) Representative PET-CT images of defect sites at 8 weeks post-implantation. (**C**) Quantitative analysis of bone area/tissue area (BA/TA); (**D**) H&E staining of the sample to illustrate the tissue structure during regenerative healing at 8 weeks. Red arrows indicate osteoblasts; black arrows point to newly formed bone (osteocyte). Scale bars represent 500 μm and 20 μm. (**E**) Quantitative analysis of bone volume/tissue volume (BV/TV) and osteoblast density (N.Ob/T.Ar) from H&E-stained sections. (**F**) Picrosirius red staining of type I collagen under light microscopy (LM) and polarized light microscopy (PLM) at 8 weeks (scale bar: 50 μm). Ot free: PCL/GelMA scaffold with BMSCs alone; WTO: PCL/GelMA scaffold with WTO + BMSCs; SOOME: PCL/GelMA scaffold with SOOME + BMSCs. Results are presented as mean ± standard deviation (*n* = 5). *P < 0.05 vs. Ot-free, ^#^*P *< 0.05 vs. WTO by one-way ANOVA.

#### CT scans of new bone formation induced by SOOME

To evaluate the effect of SOOME on bone defect repair, CT scans were used to monitor new bone formation dynamically at 4 and 8 weeks after implantation. At 4 weeks, substantial modular bone formation was observed in both the WTO and SOOME groups (Supplementary Figure S3A, right), whereas only minimal newly formed bone was seen in the Ot-free group (Supplementary Figure S3A, left). Quantitative analysis showed that the bone area/tissue area (BA/TA) in the SOOME group reached 14.22%, significantly higher than 6.1% in the Ot-free group and 8.17% in the WTO group (Supplementary Figure S3B). By week 8, BA/TA values had increased in all groups compared with week 4. Notably, the SOOME group exhibited the strongest osteogenic capacity (Figure 3B), with BA/TA increasing to 52.3%, which was 5.11-fold and 2.62-fold higher than that of the Ot-free group (10.23%) and the WTO group (20.0%), respectively (Figure 3C). These results indicated that SOOME promoted the repair of critical-size bone defects more effectively than the Ot-free and WTO groups.

#### Bone histomorphometric analysis of SOOME-formed bone

Morphometric analysis revealed that the SOOME group exhibited significant osteogenic advantages. At 4 weeks, the BV/TV in the SOOME group reached 22.73%, substantially increased relative to the Ot-free group (8.82%) and the WTO group (15.06%); moreover, the newly formed tissue in the Ot-free group lacked typical bone structural features (Supplementary Figure S3C and D). By week 8, the osteogenic advantage of the SOOME group became even more pronounced (Figure 3D): BV/TV of the newly formed bone increased to 58.40%, which was 3.2-fold and 1.93-fold higher than that of the Ot-free group (18.25%) and the WTO group (30.31%), respectively (Figure 3E). The newly formed bone not only exhibited typical compact structural features, including osteocytes within the mineralized matrix and osteoblasts arranged in an orderly manner at the margins (enlarged view in Figure 3D), but also showed good integration with the host bone. In contrast, the newly formed bone in the other two groups had a loose structure and lacked the structural integrity observed in the SOOME group. Osteoblast density in the SOOME group (3.90 × 10^2^/mm^2^) was significantly higher than that in the Ot-free group (1.35 × 10^2^/mm^2^) and the WTO group (1.83 × 10^2^/mm^2^), representing 2.9-fold and 2.1-fold increases, respectively (Figure 3E), confirming the dual advantages of the SOOME group in promoting both the quality and quantity of bone regeneration.

#### Osteoblast activity in SOOME-induced bone

Osteoblasts are the primary functional cells responsible for bone matrix formation, secreting collagen and promoting its mineralization. Picrosirius red staining revealed the structural characteristics and spatial arrangement of Type I collagen deposited by osteoblasts. At 4 weeks after implantation, the SOOME group exhibited the most pronounced increase in collagen fibers, which were tightly and orderly arranged; the WTO group followed, while the Ot-free group showed sparse and disorganized collagen fibers (Supplementary Figure S3E). By 8 weeks, collagen deposition had increased significantly in all three groups; however, the SOOME group exhibited the most closely resembling that of native bone tissue (Figure 3F). Under light microscopy, the SOOME group showed dense red staining, and under polarized light, it exhibited typical red–yellow birefringence, indicating highly ordered collagen fiber arrangement. In contrast, the WTO group exhibited moderate collagen deposition, whereas the Ot-free group showed sparse, disorganized deposition. Therefore, SOOME induced stronger osteogenic potential in osteoblasts compared to the WTO and Ot-free groups.

### Osteoclastogenesis by SOOME

#### Osteoclast activity in SOOME-induced bone

To determine whether SOOME induces physiological bone resorption alongside bone formation, osteoclastogenesis was examined via TRAP staining. As shown in Figure 4A and B, the SOOME group exhibited a marked increase in osteoclasts after implantation. At 4 weeks, the osteoclast surface area density (Oc.S/T.Ar) reached 10.93 μm/mm^2^, significantly higher than that of the WTO (4.07 μm/mm^2^) and the Ot-free group (0.42 μm/mm^2^). By week 8, Oc.S/T.Ar in the SOOME group had further increased to 21.78 μm/mm^2^ (a 99.27% increased from week 4), and osteoclasts were observed growing and interconnecting within the newly formed bone, whereas osteoclasts in the WTO group (9.07 μm/mm^2^) and the Ot-free group (5.43 μm/mm^2^) remained scattered (Figure 4A and B).

**Figure 4:**
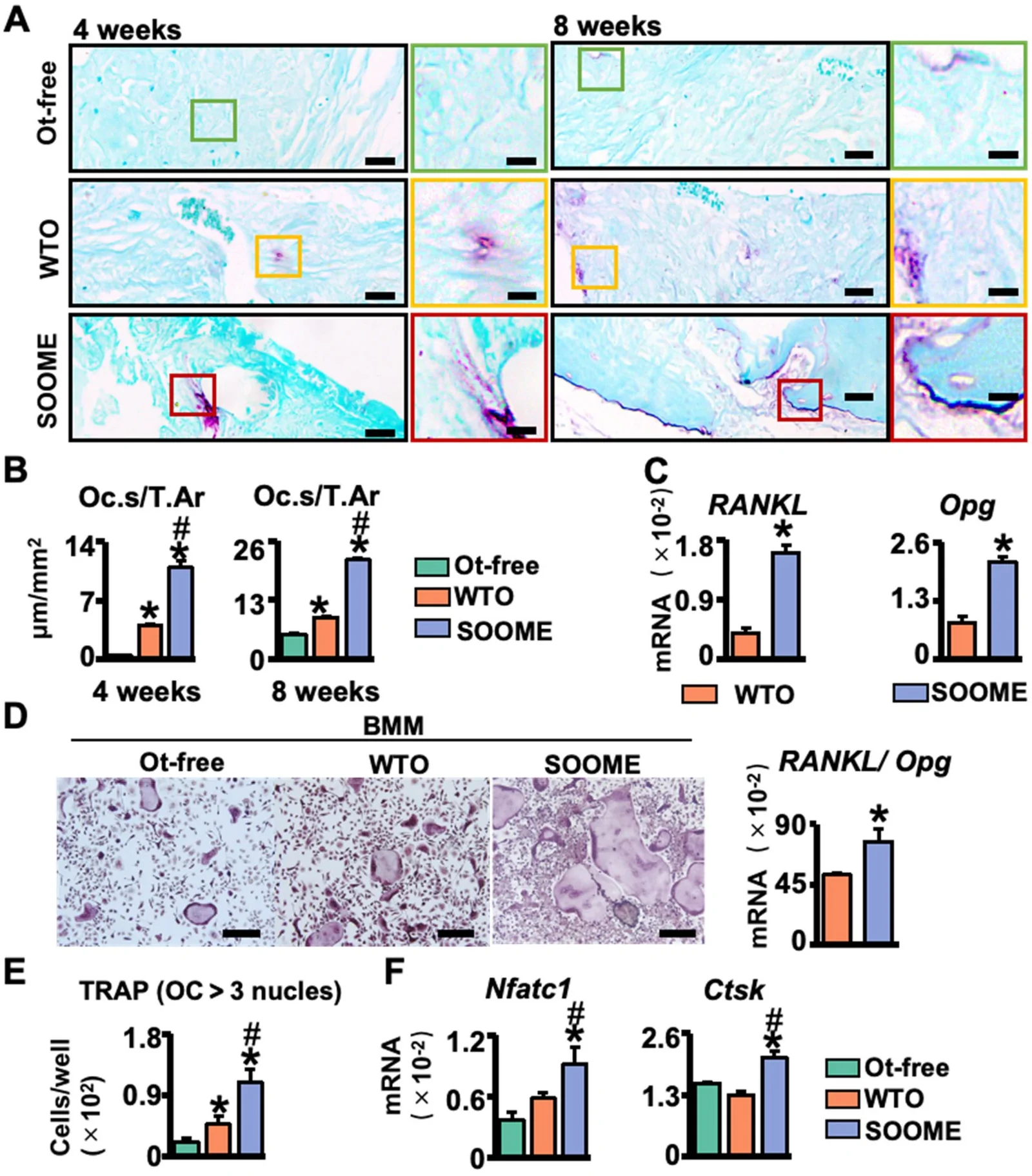
Osteoclastogenesis by SOOME. (**A**) TRAP staining of paraffin sections post-implantation; red indicates TRAP-positive osteoclasts (scale bars: 50 μm and 20 μm). (**B**) Quantitative analysis at 4 and 8 weeks: Proportion of osteoclast surface area relative to total tissue area (Oc.S/T.Ar). Representative histological sections were analyzed per animal (*n* = 5). (**C**) qPCR detects pro-osteoclastogenic cytokines. (**D**) TRAP staining of BMMs co-cultured with WTO and SOOME for 5–7 days (scale bar = 100 μm) and counted osteoclasts with more than 3 nuclei (**E**). (**F**) qPCR detects osteoclast differentiation marker genes after 5–7 days of culture. *n* = 3 for *in vitro* assays. *In vivo*: Ot free: PCL/GelMA scaffold with BMSCs alone; WTO: PCL/GelMA scaffold with WTO + BMSCs; SOOME: PCL/GelMA scaffold with SOOME + BMSCs. *In vitro*: Ot-free: BMM alone; WTO: WTO co-cultured with BMM; SOOME: SOOME co-cultured with BMM. Data were expressed as mean ± SD. *P < 0.05 vs. Ot-free, ^#^*P *< 0.05 vs. WTO by one-way ANOVA.

#### RANKL/OPG expression of SOOME

qPCR analysis of pro-osteoclastogenic cytokines (Figure 4C) revealed that the SOOME group exhibited the highest RANKL mRNA levels, which were 3.97-fold higher than those in the WTO group. Concurrently, OPG expression increased by 2.68-fold, and the RANKL/OPG ratio rose by 1.48-fold, indicating that SOOME promoted a microenvironment favorable for osteoclast differentiation.

#### Osteoclastogenesis by SOOME *in vitro*

To evaluate SOOME’s role in osteoclast differentiation, WTO and SOOME were co-cultured with BMMs, respectively, and all groups were treated with recombinant M-CSF and RANKL. TRAP staining and quantification (Figure 4D and E) revealed that the number of SOOME TRAP^+^ cells was 4.8-fold and 2.3-fold higher than that in the Ot-free and WTO groups, respectively, and that SOOME induced the formation of more large, multinucleated osteoclasts. qPCR analysis showed that osteoclast-specific genes were significantly upregulated in the SOOME group (Figure 4F); the *Nfatc1* mRNA was 1.58-fold and 2.46-fold higher than in the Ot-free and WTO groups, respectively, and the *Ctsk* mRNA levels were 1.61-fold and 1.35-fold higher. These results demonstrated the pro-osteoclastogenic potential of SOOME. Collectively, these findings from both cellular distribution and molecular regulation perspectives demonstrated that SOOME, by optimizing the bone microenvironment, not only promoted early bone resorption but also supported subsequent bone formation.

### Potential of angiogenesis by SOOME

#### *In vivo* angiogenesis by SOOME

Angiogenesis analysis indicated that SOOME had a significant vascularization. H&E staining at 4 weeks after implantation (Supplementary Figure S4A and B) showed that vascular density in the SOOME group was 4.31 vessels/mm^2^, which was 2.33 and 1.74 times higher than that in the Ot-free group (1.19 vessels/mm^2^) and the WTO group (2.79 vessels/mm^2^). At 8 weeks, the vascular density in the SOOME group (N.Vessel/T.Ar) reached a maximum of 8.24/mm^2^, which was 3.26-fold and 1.52-fold higher than that of the Ot-free group (2.53/mm^2^) and the WTO group (5.42/mm^2^), respectively (Figure 5A and B). Immunofluorescence staining further confirmed these findings: the SOOME group exhibited significantly enhanced double-positive signals for CD31 (red fluorescence) and Endomucin (green fluorescence), indicating the formation of a bone-specific H-type mature vascular network (Figure 5C and D). These new blood vessels were primarily distributed in areas of active bone regeneration and exhibited a clear spatiotemporal correlation with osteogenic activity. This pro-angiogenic effect not only provided the necessary oxygen and nutritional support for bone tissue repair but also delivered key growth factors and cellular components, reflecting the unique “vascular-osteogenic coupling” regulatory capacity of SOOME, which may be a key mechanism underlying its significant enhancement of bone regeneration.

**Figure 5:**
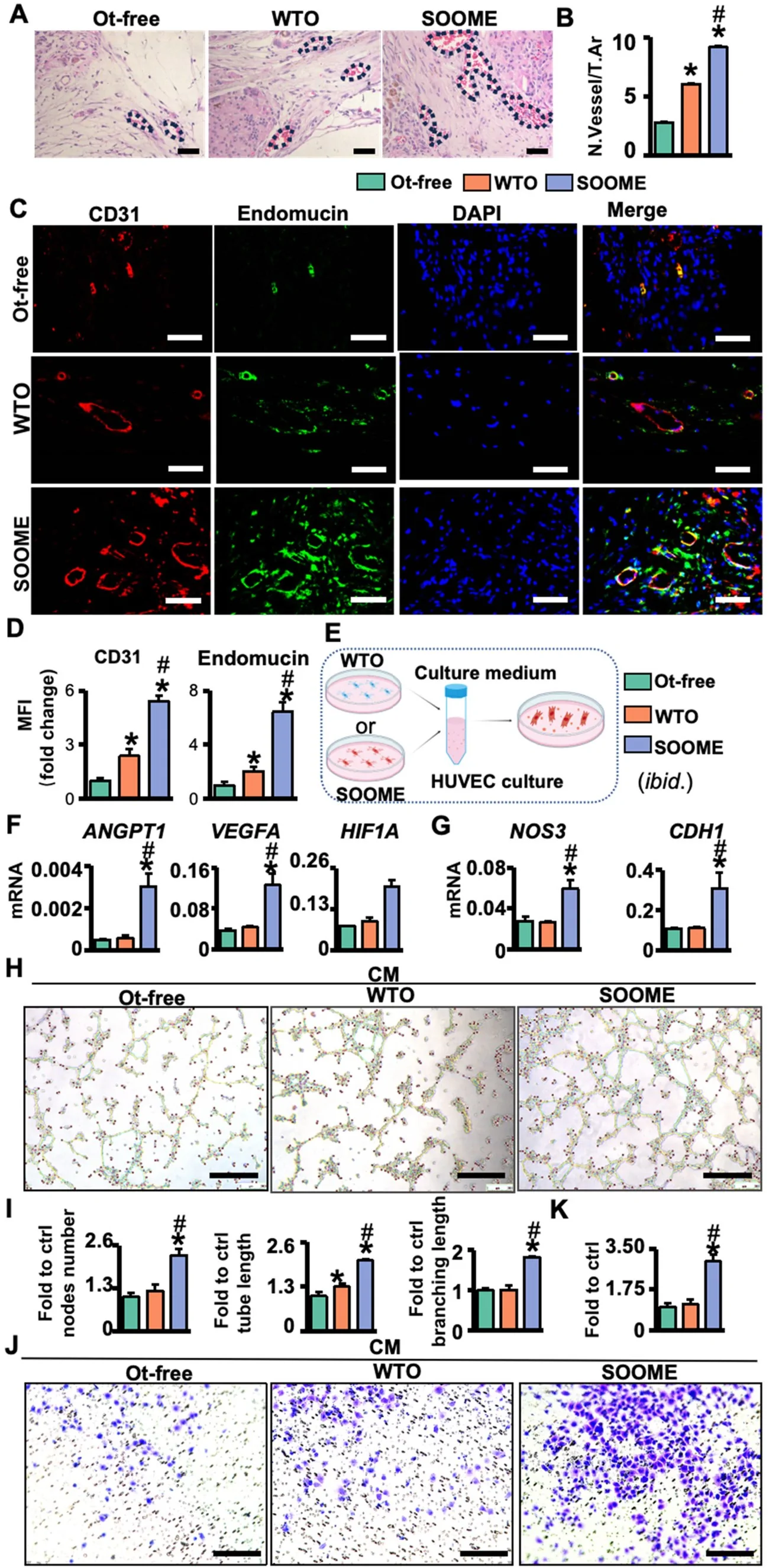
Potential of angiogenesis by SOOME. (**A**) H&E staining of vascular morphology at 8 weeks after implantation (scale bar = 20 μm). (**B**) Quantification of the vessel density, expressed as the number of vessels per unit tissue area (N.Vessel/T.Ar). (**C**) Evaluation of *in vivo* H-type angiogenesis in bone defect regions using CD31 and Endomucin immunofluorescence staining. Scale bar = 20 μm. (**D**) Quantification of relative mean fluorescence intensity (MFI) of CD31 and Endomucin. Data were normalized to the Ot-free group (set as 1). (**E**) Flowchart showing the treatment of HUVECs with or without WTO or SOOME-conditioned medium (CM). (**F, G**) qPCR analysis of the expression of angiogenic factors and mRNA associated with vascular function in the presence and absence of conditioned medium. (**H, I**) Images of tubular structures formed by HUVECs on Matrigel (scale bar = 100 μm) (**H**), and (**I**) statistical analysis of the number of nodules, branch length and total length. (**J, K**) Endothelial cell migration assay in the transwell system: (**J**) migration images (scale bar = 100 μm) and (**K**) migrated HUVECs counts after 48 hours of treatment with conditioned medium. Sections containing new bone formation were selected from each mouse for measurement. (*n* = 3 independent experiments for *in vitro* assays; *n* = 5 mice per group *in vivo*). *In vivo*: Ot free: PCL/GelMA scaffold with BMSCs alone; WTO: PCL/GelMA scaffold with WTO + BMSCs; SOOME: PCL/GelMA scaffold with SOOME + BMSCs. *In vitro*: Ot-free: HUVEC alone; WTO: HUVEC treated with WTO-CM; SOOME: HUVEC treated with SOOME-CM. Data are presented as mean ± standard deviation. *P < 0.05 compared to the Ot-free group; ^#^*P *< 0.05 compared to the WTO group, using one-way ANOVA.

#### Effects of SOOME conditioned medium on angiogenesis *in vitro*

To evaluate the pro-angiogenic effects of SOOME, the effects of conditioned medium (CM) from the three groups on HUVECs were investigated (Figure 5E). qPCR analysis showed that SOOME specifically upregulated the HUVEC’s the expression of key angiogenic genes *ANGPT1* and *VEGFA* (Figure 5F), as well as vascular function-related genes *HIF1A*, *NOS3* and *CDH1* (Figure 5G). Vascular network formation assays (Figure 5H and I) revealed that the numbers of vascular nodules, total length and branch length formed by HUVECs induced by SOOME were 2.96-fold, 2.21-fold and 1.83-fold higher than those in the Ot-free group, respectively, and 1.90-fold, 1.58-fold and 1.80-fold higher than those in the WTO group. Transwell assays (Figure 5J and K) showed that the number of endothelial cells migrating in response to SOOME-CM was 2.96-fold and 2.65-fold higher than that in the Ot-free and WTO groups, respectively. Collectively, these results confirmed that SOOME possessed robust pro-angiogenic properties at both the cellular and molecular levels.

### Pro-neurogenic potential of SOOME

#### *In vivo* neurogenesis assay by SOOME

Neural regeneration analysis revealed the unique neurotrophic properties of SOOME. Immunofluorescence quantification displayed that β3-tubulin and NeuN were higher in the SOOME group than in the Ot-free and WTO groups (Figure 6A). Quantitative analysis further revealed that the relative fluorescence intensity of β3-tubulin in the SOOME group was 2.4-fold and 5.6-fold higher than that in the Ot-free and WTO groups, respectively; while the relative fluorescence intensity of NeuN was 2.0-fold and 6.4-fold higher, respectively (Figure 6B).

**Figure 6:**
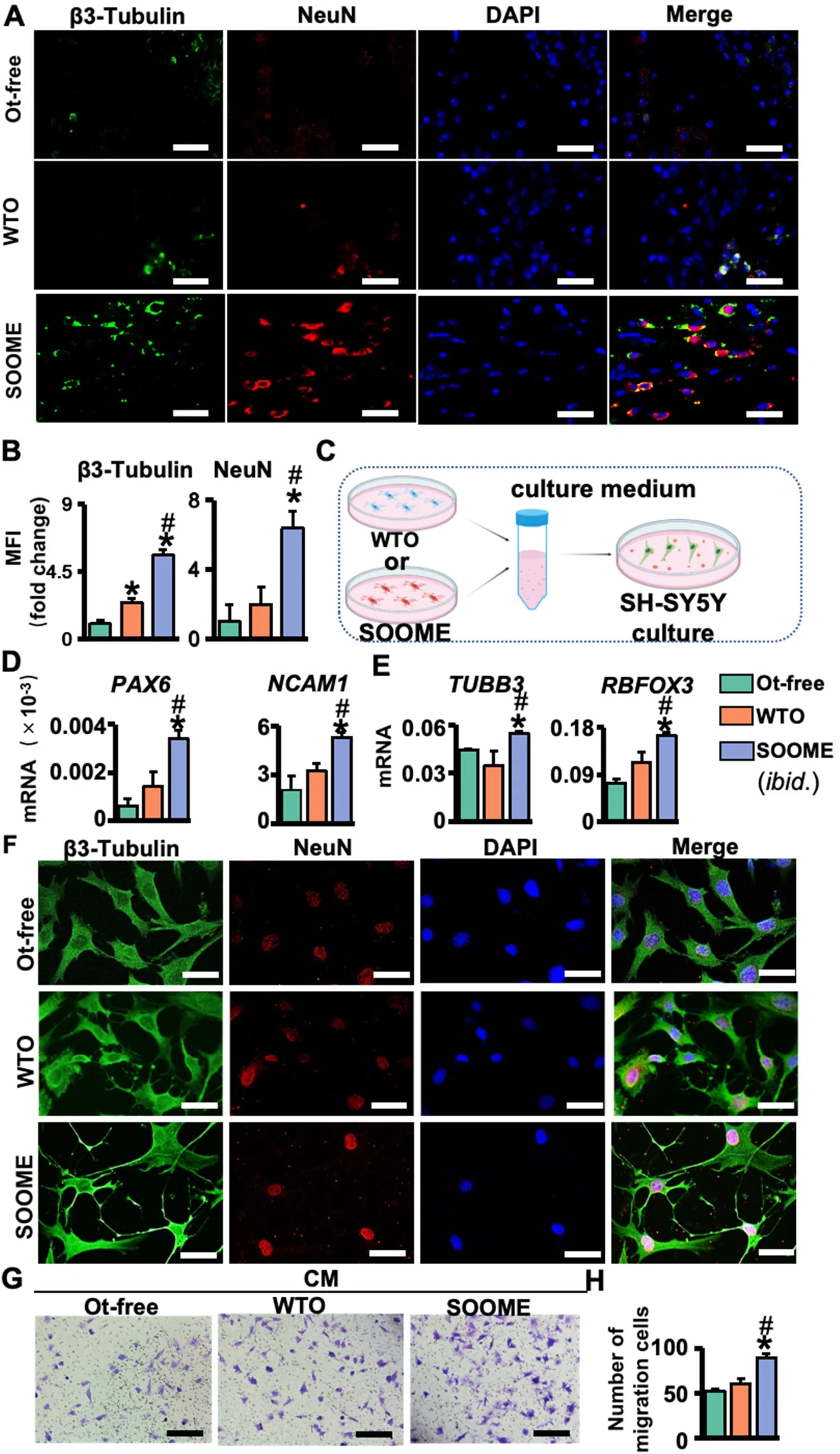
Neurogenesis by SOOME. (**A**) *In vivo* neurogenesis in the bone defect regions was assessed using β3-tubulin and NeuN immunofluorescence staining. Scale bar = 20 μm. (**B**) Quantification of relative mean fluorescence intensity (MFI) of β3-tubulin and NeuN in the calvarial defect region at 8 weeks post-implantation. Data were normalized to the Ot-free group (set as 1). (**C**) SH-SY5Y cell culture flowchart: SH-SY5Y treated by CM from WTO and SOOME. (D-E) qPCR detection of neural-related genes (**D**) and neuro-marker genes (**E**) in SH-SY5Y treated with/without WTO or SOOME CM. (**F**) Immunofluorescence of β3-tubulin and NeuN showed SH-SY5Y *in vitro*. Scale bar = 20 μm. (**G, H**) SH-SY5Y cell migration assay in a transwell system: (**G**) Migration images and (**H**) cell counts after 48 hours of conditioned medium treatment (scale bar = 100 μm); sections containing new bone formation were selected from each mouse for measurement; five mice per group were analyzed. *In vivo*: Ot free: PCL/GelMA scaffold with BMSCs alone; WTO: PCL/GelMA scaffold with WTO + BMSCs; SOOME: PCL/GelMA scaffold with SOOME + BMSCs. *In vitro*: Ot-free: SH-SY5Y alone; WTO: SH-SY5Y treated with WTO-CM; SOOME: SH-SY5Y treated with SOOME-CM. Data are presented as mean ± standard deviation. *P < 0.05 compared with the Ot-free group; ^#^*P *< 0.05 compared with the WTO group, using one-way ANOVA.

#### *In vitro* neurogenesis by SOOME

To further validate the neural regulation effects of SOOME, we treated SH-SY5Y with CM from WTO and SOOME *in vitro* (Figure 6B). qPCR analysis revealed that treatment with SOOME CM markedly elevated the SH-SY5Y’s expression of the neurodifferentiation-related genes *NCAM1* and *PAX6* (Figure 6D) as well as the synaptic and mature neuron markers *TUBB3* and *RBFOX3* (Figure 6E). Immunofluorescence staining further confirmed that the SOOME group exhibited stronger expression of β3-tubulin and NeuN proteins and formed more synapses (Figure 6F, Figure S8A). Transwell assays further confirmed that the number of migrating SH-SY5Y cells induced by SOOME-conditioned medium was 1.72-fold and 1.48-fold higher than that in the Ot-free and WTO groups, respectively (Figure 6G and H). These results demonstrated, at the molecular and cellular levels, that SOOME not only promoted neural regeneration *in vivo* but also neuronal differentiation *in vitro*.

### Transcriptomic analysis of SOOME

To determine how SOOME influenced osteogenesis of BMSCs, we performed mRNA sequencing on WTO and SOOME samples. Sample correlation analysis verified high biological reproducibility (Figure 7A). Based on this, we identified 3,077 distinguished genes in the SOOME group relative to the WTO group, of which 2,354 were upregulated, and 723 were downregulated (Figure 7B). To further explore the biological functions of these different genes, we performed KEGG and GO pathway and term enrichment analyses (Supplementary Figure S5A and B). KEGG pathway analysis displayed the top 20 pathways in SOOME (Supplementary Figure S5A); the increased genes were primarily expressed in pathways associated with cell adhesion molecules, neuroactive ligand-receptor interactions, axon guidance, the Hippo signaling pathway, calcium signaling, extracellular matrix–receptor interactions and the Wnt signaling pathway. These results suggested that Wnt activation enhanced osteocyte biological activity and influenced the microenvironment by regulating processes such as neural signal transduction, vascular development and bone remodeling. Meanwhile, GO enrichment analysis revealed the top significantly enriched GO terms in the SOOME group (Supplementary Figure S5B), primarily involving homophilic cell adhesion, plasma membrane adhesion molecules, membrane potential regulation, regulation of monovalent ion transport across membranes, synaptic assembly, neurotransmitter transport and synaptic signal transduction, further supporting that Wnt activation influenced the microenvironment through neural signaling, ion transport and synaptic organization. These results also suggested that SOOME may integrate into the bone microenvironment and help regulate bone tissue regeneration through neuro-osseous coupling mechanisms.

**Figure 7:**
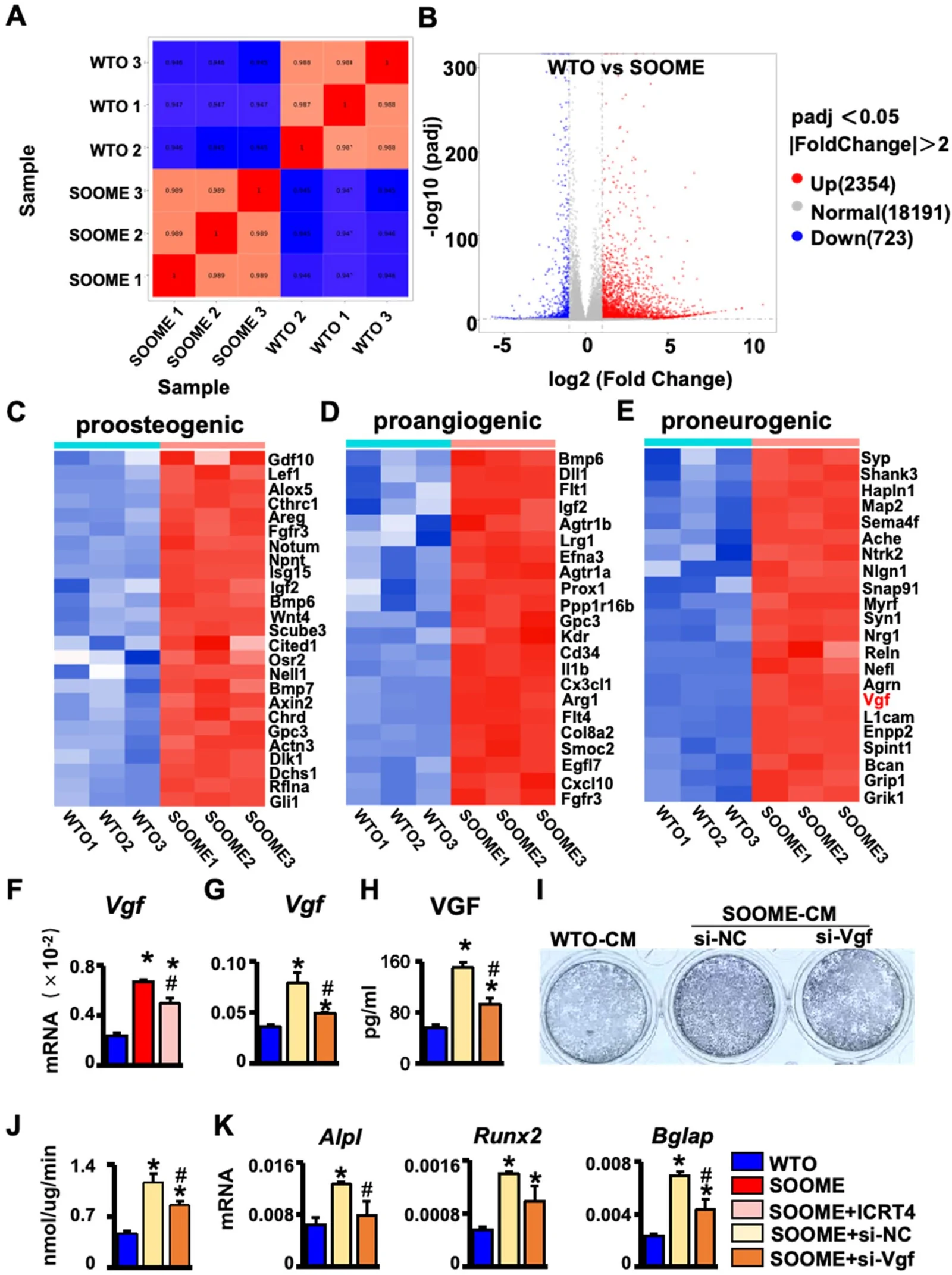
Transcriptomic analysis of SOOME. (**A**) Sample correlation in gene expression patterns. (**B**) Number of DEGs in different comparison groups. (**C**) Heatmap showing gene expression promoting osteogenesis (**D**), angiogenesis (**E**) and neurogenesis in the WTO and SOOME groups. (**F**) *Vgf* mRNA expression levels in osteocytes from the WTO, SOOME and SOOME + ICRT4 groups; (**G**) *Vgf* mRNA expression levels in osteocytes from the WTO, SOOME+ si-NC and SOOME + si-*Vgf* groups. (**H**) VGF secretion levels in the cell culture supernatant were quantified by ELISA across the WTO, SOOME + si-NC and SOOME + si-*Vgf* groups. (**I, J**) Alkaline phosphatase staining and quantitative analysis of BMSCs treated with the WTO, SOOME+ si-NC and SOOME + si-*Vgf* groups; (**K**) Osteogenic marker genes in BMSCs treated with conditioned medium from the WTO, SOOME+ si-NC and SOOME + si-*Vgf* groups. Ot-free: BMSC alone; WTO: BMSC treated with WTO-CM; SOOME: BMSC treated with SOOME-CM. Data are presented as mean ± SD (*n* = 3). *P < 0.05 vs. WTO group; ^#^*P *< 0.05 vs. SOOME/SOOME + si-NC, group, using one-way ANOVA.

Additionally, the heatmap showed that the expression of several representative bone-formation genes was upregulated in the SOOME group (Figure 7C), corroborating the effect of SOOME on pro-osteogenic and mineralization in BMSCs. Genes associated with angiogenesis and neurogenesis also showed an upregulated trend in the SOOME group (Figure 7D and E), suggesting that SOOME may create a microenvironment conducive to osteogenic differentiation, angiogenesis and neurogenesis.

### Functional validation of SOOME-mediated upregulation of *Vgf*

In recent years, the interaction between bone tissue and the nervous system has garnered increasing attention. Through differential gene screening, we identified the nerve growth factor-induced protein Vgf as a significantly upregulated gene in SOOME. Vgf not only participates in neuroprotection and axonal regeneration but also regulates osteogenic differentiation. To investigate the role of *Vgf* in the bone-nerve regulatory network, we conducted a series of *in vitro* functional experiments.

qPCR results showed that *Vgf* was significantly upregulated in the SOOME, and treatment with a Wnt inhibitor partially reversed *Vgf* expression (Figure 7F). To determine whether *Vgf* mediated the osteogenic effects of SOOME, we used siRNA to knock down *Vgf* expression and confirmed the effective knockdown of VGF at both the gene and protein levels (Figure 7G and H). ALP staining, biochemical activity quantification and osteogenic marker gene analysis consistently indicated that silencing *Vgf* in the context of Wnt activation (SOOME + si-*Vgf* group) effectively reversed the osteogenic effect (Figure 7I–K; Supplementary Figure S6), suggesting that *Vgf* may be one of the important mediators of Wnt signaling-induced osteogenic differentiation. Furthermore, we investigated whether *Vgf* participated in the regulation of neuronal differentiation by SOOME. Immunofluorescence staining revealed that the CM from the SOOME group significantly increased the neuronal markers β3-tubulin and NeuN in SH-SY5Y cells, whereas the CM from the SOOME + si-*Vgf* group partially reduced the fluorescence intensity of these markers (Figure 8A; Supplementary Figure S9A). Cell migration assays further demonstrated that *Vgf* knockdown attenuated the pro-migratory effect of SOOME + si-NC-CM on SH-SY5Y cells (Figure 8B and C). To validate the neuro-osteogenic interaction, we co-cultured SH-SY5Y cells pre-treated with the aforementioned CM with BMSCs at a 1:4 ratio (Figure 8E). Compared with the WTO group, SH-SY5Y cells treated with SOOME + si-NC -CM enhanced the osteogenic activity of BMSCs, whereas the SOOME + si-*Vgf* group exhibited a partial reversal (Figure 8F–H), suggesting that SOOME may promote the neural differentiation of SH-SY5Y cells through Vgf secretion, thereby indirectly enhancing osteogenic differentiation.

**Figure 8:**
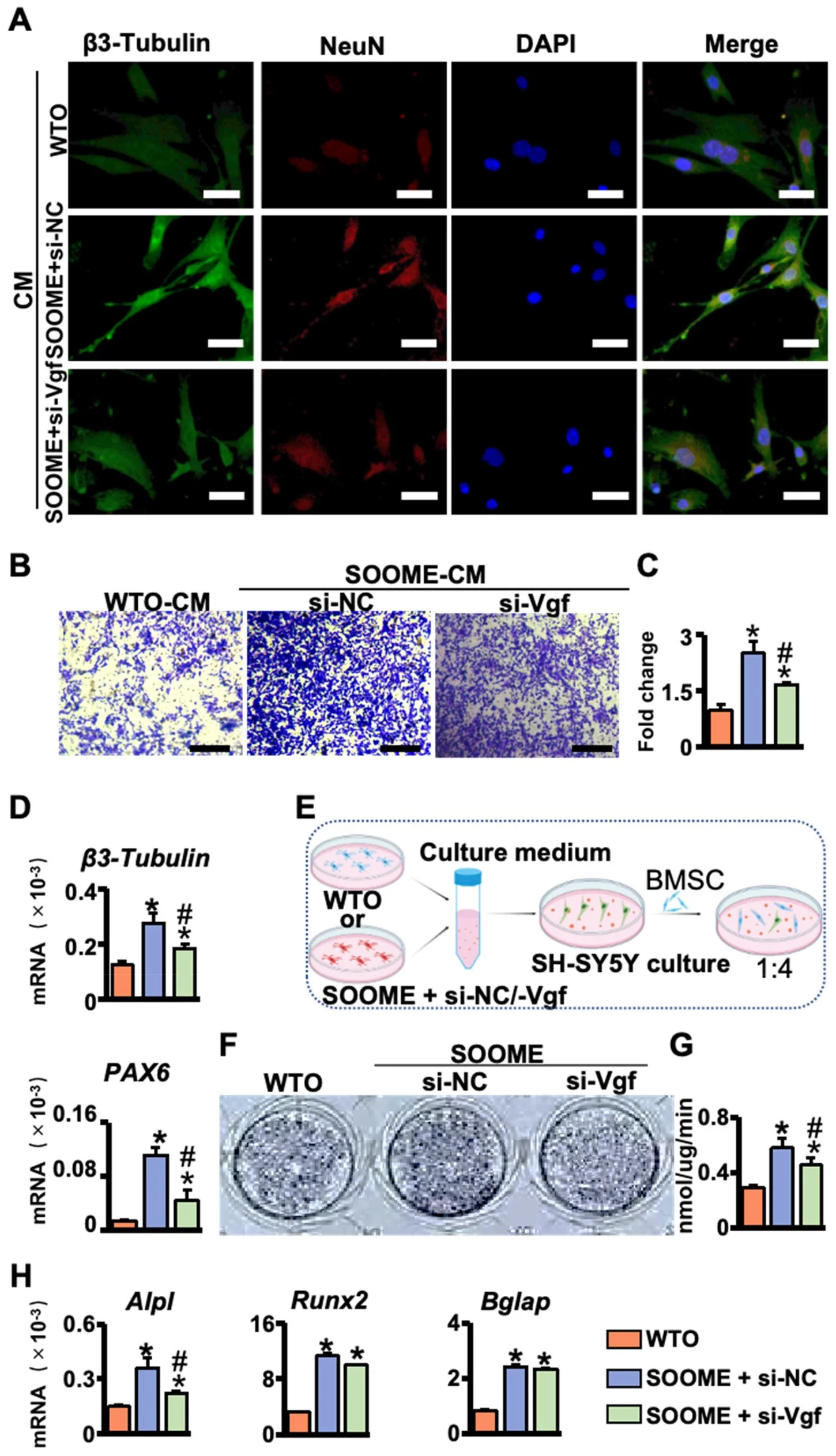
Functional validation of the *Vgf* in SOOME activity. (**A**) Immunofluorescence analysis of β3-tubulin and NeuN protein expression in SH-SY5Y pre-treated with CM of the WTO, SOOME + si-NC and SOOME + si-*Vgf* groups (scale bar = 20 μm); (**B, C**) transwell assay to detect the role of above CM on SH-SY5Ys’ migration ability (scale bar = 100 μm); (**D**) qPCR analysis of mRNA of *TUBB3* and *PAX6* in SH-SY5Y cells treated with above CM ; (**E**) Flowchart of the co-culture process between SH-SY5Y incubated with above CM and BMSCs. (**F, G**) AP staining and quantitative analysis of BMSCs co-cultured with SH-SY5Y exposed to the various CM. (**H**) qPCR detection of osteogenic marker gene in BMSCs within the co-culture system. Data are presented as mean ± SD (*n* = 3). *P < 0.05 vs. WTO group; ^#^*P *< 0.05 vs. SOOME + si-NC group; one-way ANOVA.

## Discussion

To achieve multisystem-coordinated bone repair, we engineered a 3D-bioprinted platform centered on Wnt-activated osteocyte osteogenic microenvironment (SOOME). This construct synergistically regulated osteogenesis, osteoclastogenesis, angiogenesis and neurogenesis *in vitro* and *in vivo*, thereby enhancing bone regeneration in a mouse critical-sized calvarial defect model. Mechanistically, transcriptomic analysis identified *Vgf* as a downstream gene that partially contributed to the direct osteogenic differentiation and indirect neuro-osteogenic coupling of SOOME.

The synergistic effect of osteogenesis, angiogenesis and neurogenesis is fundamental to bone repair. However, existing strategies frequently rely on complex multicellular co-cultures or the exogenous delivery of growth factors, peptides or drugs [13, 58–60]. The stability of multi-cell coexistence and the uncontrollable release of factors pose challenges to production consistency, regulatory approval and clinical scaling. Our work distinguishes itself from these strategies [4] through a SOOME-centered system using only two bone-lineage cell types: Wnt-activated osteocytes (SOOME) create an osteogenic microenvironment associated with osteogenesis, osteoclastogenesis, angiogenesis and innervation, while BMSCs serve as osteogenic effectors. This design offers a simpler and more controllable alternative to multi-cellular or growth-factor-based strategies, without requiring complex cell culture conditions or external supplementation.

Rather than the conventional “anti-resorption” strategy [61–63], SOOME reveals a dynamic osteoclastic-osteogenic coupling mechanism that accelerates bone integration. Osteoclasts, recognized as bone-resorbing cells, dominate the degradation of the bone matrix [64,65]; however, emerging studies have revealed that they coordinate angiogenesis, stem cell chemotaxis and osteogenic differentiation by secreting coupling factors such as PDGF-BB, TGF-β and IGF-1 [17, 66–68]. In this study, SOOME, which highly expresses OPG, also exhibits higher RANKL levels, resulting in an increased RANKL/OPG ratio, which was associated with a marked increase in osteoclast surface area at the defect site. Remarkably, despite pronounced osteoclastic activity, the SOOME group also yielded superior volumes of newly regenerated bone at 4 and 8 weeks. This “paradoxical” phenomenon underscores that osteoclasts may collaboratively promote angiogenesis and osteogenic differentiation by secreting coupling factors such as PDGF-BB and TGF-β [17, 66–69], although the contribution of these pathways remains to be explicitly confirmed.

Mechanistically, building on our previous research, this study further elucidates the complexity of the SOOME osteogenic effects—a process involving the synergistic regulation of multiple factors, in which Vgf partially contributed to osteoneural interaction. In recent years, the regulatory role of neurogenesis in bone repair has gained appreciation [70]. In addition to neurotrophic factors such as BDNF, NGF, substance P, CGRP and VIP influencing bone formation [71–73], Koh *et al.* recently discovered that Vgf and its derivative peptide AQEE-30 can stimulate osteoblast differentiation via the C3a receptor [43]. However, the regulatory relationship between Wnt signaling and *Vgf* in osteocytes remains unclear [74]. As a neuropeptide precursor induced by NGF, BDNF and others, Vgf has been suggested in the literature to potentially interact with the Wnt pathway via BDNF [75,76]. Our data indicated that Wnt signaling upregulates *Vgf* expression in osteocytes, and that *Vgf* partially contributed to the direct osteogenic effects of SOOME as well as its ability to promote neuronal differentiation, consistent with reports that Vgf acts as a downstream effector of BDNF in promoting neurogenesis [76].

To evaluate the functional significance of *Vgf*, we performed Vgf knockdown experiments. Effective VGF knockdown (Figure 7G and H) only partially reversed the osteogenic effect, but almost eliminated the neural effect, suggesting that *Vgf* exerts differential effects in SOOME-mediated osteogenic and neural processes (Figures 7H–J and 8A–D)—serving as a primary driver of neural differentiation but only an auxiliary regulator of osteogenesis. Consequently, we propose that *Vgf* modulates bone formation through dual pathways: the direct pathway and the indirect pathway mediated through the neuro-osteogenic feedback loop (Figure 8E–H). The incomplete reversal of the osteogenic phenotype upon *Vgf* knockdown further suggests the involvement of other factors secreted by SOOME. Indeed, secretome profiling (Supplementary Figure S7) revealed a rich composition of osteogenic (*Col9a1*/*Col9a2*, *Igf2*, *Wnt6*, *Dkkl1*, *Mstn*), angiogenic (*Dhh*, *Egflam*, *A2m*, *Spock1*, *Lipg*) and neurogenic factors (*Pnoc*, *C1ql1*, *C1ql4*, *Ncan*, *Sbspon*, *Nell2*, *Chga*, *Klk8*, *Vgf*). Notably, *Igf2*, *Nell2* and *Vgf* emerged as dual-function factors capable of promoting both osteogenesis and neurogenesis. These findings collectively indicate that the pro-regenerative activity of SOOME does not rely on a single dominant factor such as *Vgf* but rather arises from a coordinated network of multiple secreted factors acting in synergy.

This multi-factor synergy is consistent with our previous work demonstrating that the osteogenic effects of osteocytic Wnt result from the coordinated action of multiple pathways, including BMP [22, 77] and Notch [40] pathways. Future research will focus on elucidating the relationship and mutual regulation between *Vgf* and these identified independent osteogenic factors to further deepen our understanding of the complex regulatory network of Wnt signaling in osteocytes.

Several limitations should be acknowledged. First, the *in vivo* survival of SOOME and BMSCs after implantation remains unclear. Lineage tracing (e.g. DiR labeling of SOOME and GFP labeling of BMSCs) will be incorporated in future studies to track the fate of these two cell types. Second, a blank scaffold group and a SOOME-alone group would provide a more complete baseline for evaluating individual contributions. Nevertheless, the primary focus of this study was to validate the necessity of Wnt-activated osteocytes for BMSC osteogenic differentiation. Future studies will include these additional controls. Third, the lack of dynamic histomorphometric data (e.g. calcein/alizarin red S double labeling) limits our ability to distinguish coupled remodeling from woven bone accumulation. Future studies will incorporate sequential fluorescent labeling to directly validate the coupled remodeling mechanism underlying SOOME-mediated bone regeneration.

Beyond these experimental design considerations, the reliance on viable cell transplantation poses a more fundamental barrier to clinical translation. We are currently exploring osteocytic decellularized matrices (DM) based on SOOME, aiming to reduce immunogenicity and enhance product stability. Concurrently, to bridge the gap between rodent studies and clinical application, our ongoing research will pivot toward a minipig model using homologous porcine osteocytes to validate the repair efficacy and safety, thereby providing critical preclinical evidence for eventual clinical translation.

In summary, we developed an orthopedic artificial bone graft using the S24 drug to generate SOOME, characterized it as having high metabolic activity and the ability to orchestrate neurovascular development. Through transcriptomic analysis, we identified that *Vgf* partially contributed to both osteogenic differentiation and neuro-osteogenic crosstalk. Additionally, with enhanced osteoclast recruitment, we observed elevated bone resorption and formation, which occurred in close spatial proximity. This discovery provides a new approach to developing “living” bone grafts with biointegrative capabilities and may offer new strategies for clinical bone defect repair.

## Conclusions

To address the growing demand for functionally enhanced seed cells and multifunctional bone grafts capable of coordinating complex tissue regeneration, this study establishes a SOOME-based platform as a multifunctional strategy for critical-sized bone defect repair. Through integrated *in vitro* and *in vivo* evaluations, SOOME was shown to accelerate the repair of critical-sized bone defects by promoting osteogenesis and osteoclast recruitment, and neurovascularization, which are essential processes in natural bone healing. The identification of *Vgf* as a partial mediator of neuro-osteogenic function offers a potential mechanistic target for future investigation. Together, these findings provide a foundation for developing functionally enhanced seed cell-based constructs and bioactive bone grafts for clinical translation.

## Supplementary Material

rbag124_Supplementary_Data
