# Supplementary material for: Wnt-activated osteocyte-driven 3D-bioprinted scaffolds orchestrate bone remodeling, angiogenesis and innervation to accelerate critical bone defect repair
Source: Regen Biomater. 2026 Jun 16;13:rbag124. doi: 10.1093/rb/rbag124 (PMC13695564; doi:10.1093/rb/rbag124)
Supplement: Supplementary file 1 [file rbag124_supplementary_data.docx]

**Wnt-activated osteocyte-driven 3D-bioprinted scaffolds orchestrate bone remodeling, angiogenesis, and innervation to accelerate critical bone defect repair**

1. **Supplementary Figures and Legends**

**Fig. S1.** ALP staining of the co-culture system (osteocytes and BMSCs) at different cell proportions. (A) Representative images of ALP staining in osteocytes and BMSCs at varying cell proportions are shown.

**Fig. S2. Macroscopic observation of scaffold implantation and retrieval.** (A) Implantation procedure: (a) Implants were placed in living subjects; (b) Scaffolds were carried out in vivo at 8 weeks (preharvest).

**Fig. S3**. Bone formation by SOOME in vivo at 4 weeks. (A) Representative PET-CT images of defect sites at 4 weeks post-implantation. (B) Quantitative analysis of bone area/tissue area (BA/TA); (C) H&E staining of transverse sections to illustrate the tissue structure during regenerative healing at 4 weeks. Scale bars represent 500 μm and 20 μm. (D) Quantitative analysis of BV/TV and osteoblast density (N.Ob/T.Ar) from H&E-stained sections. (E)Picrosirius red staining of type I collagen under light microscopy (LM) and polarized light microscopy (PLM) at 4 weeks (scale bar: 50 μm). Results are presented as mean ± standard deviation (n = 5). ^*^*P* < 0.05 vs. Ot-free, ^#^*P* < 0.05 vs. WTO by one-way ANOVA.

**Fig. S4**. Potential of angiogenesis by SOOME at 4 weeks. (A) HE staining of vascular morphology at 4 weeks after implantation (scale bar: 20 μm). (B) Quantitative assessment of the vessel density, expressed as N.Vessel/T.Ar. Results are presented as mean ± standard deviation (n = 5). ^*^*P* < 0.05 vs. Ot-free, ^#^*P* < 0.05 vs. WTO by one-way ANOVA.

**Fig. S5**. KEGG and GO enrichment analysis. (A) KEGG and (B) GO enrichment analysis in the SOOME group compared with the control group. The top 20 significantly enriched pathways/terms are shown based on the adjusted $P$-value. n = 3.

**Fig. S6**. ALP staining of the BMSC treated with different groups conditioned medium. (A) Representative images of ALP staining in BMSCs.

**Fig. S7.** Identification of top upregulated secreted factors in SOOME. (A) Heatmap showing the top 20 secretion factors related to osteogenesis, neurogenesis, and angiogenesis in WTO - and SOOME.

**Fig. S8.** Quantification of relative mean fluorescence intensity (MFI) of β3-tubulin and NeuN in differentiated SH-SY5Y cells. (A) In vitro quantification. SH-SY5Y cells were treated with conditioned medium (CM) from different groups. Quantification of relative MFI of β3-tubulin and NeuN. Data were normalized to the Ot-free group (set as 1). Results are presented as mean ± SD (n = 3). ^*^*P* < 0.05 vs. Ot-free group; ^#^*P* < 0.05 vs. WTO group.

**Fig. S9.** Quantification of β3-tubulin and NeuN fluorescence intensity in SH-SY5Y cells. (A) Cells were treated with CM from WTO, SOOME+si-NC, or SOOME + si-Vgf. Fluorescence intensity was normalized to the WTO group (set as 1). Data are mean ± SD (n = 3). ^*^*P* < 0.05 vs. WTO; ^#^*P* < 0.05 vs. SOOME+ si-NC.

**Fig. S10.** Effect of S24 on osteogenic differentiation of BMSCs. (A) ALP staining of BMSCs after S24 treatment; (B) Detection of osteogenic differentiation genes in BMSCs after S24 treatment. **P* <0.05 vs. DMSO by *t* test.

3. **Supplementary Tables**

**Table S1** Antibodies for Immunostaining

| **Name** | **Company** | **Cat.No** | **Concentration** |
| --- | --- | --- | --- |
| Anti-CD31 Antibody | abcam | ab281583 | 1:200 |
| Anti-Endomucin Antibody | abcam | ab104224 | 1:200 |
| Anti-NeuN Antibody | abcam | EPR12763 | 1:200 |
| Anti-β3-Tubulin Antibody | sigma-aldrich | T8660 | 1:200 |
| Goat Anti-Rabbit IgG H&L (Alexa Fluor® 488) | abcam | ab150077 | 1:200 |
| Goat Anti-Mouse IgG, Peroxidase - Conjugated, H+L | biosharp | BL001A | 1:300 |

**Table S2** Sequences of Primers used for RT-qPCR (mouse)

| Primer | | Forward | **Reverse** |
| --- | --- | --- | --- |
| *Gapdh* | GCACAGTCAAGGCCGAGAAT | | GCCTTCTCCATGGTGGTGAA |
| *Axin2* | TGCAGGAGGCGGTACAGTTC | | GCTGGAAGTGGTAAAGCAGCTT |
| *Bmp4* | GAGGAGTTTCCATCACGAAGA | | GCTCTGCCGAGGAGATCA |
| *Smad6* | AAGATGCTGAAGCCGTTGGT | | CGAACTCCAGTATCTCCGCTTT |
| *Lef1* | TACCCCAGCCAGTGTCAACA | | TCCATGATAGGCTTTGATGACTTTC |
| *Alpl* | CACGGCGTCCATGAGCAGAAC | | CAGGCACAGTGGTCAAGGTTGG |
| *Runx2* | CCGGTCTCCTTCCAGGAT | | GGGAACTGCTGTGGCTTC |
| *Osx* | CCCTTCTCAAGCACCAATGG | | AAGGGTGGGTAGTCA TTTGCATA |
| *Bglap* | CAGCGGCCCTGAGTCTGA | | GCCGGAGTCTGTTCACTACCTTA |
| *Dmp-1* | CATTCTCCTTGTGTTCCTTTGG | | TCAGTATTGTGGTATCTGGCAACT |
| *Sost* | CGTGCCTCATCTGCCTACTTGTG | | CCGGTTCATGGTCTGGTTGTTCTC |
| *RANKL* | CATGACGTTAAGCAACGG | | AGGGAAGGGTTGGACA |
| *Opg* | CGGACAGCTGGCACACCAG | | CTCACACACTCGGTTGTGGG |
| *Rank* | TGAGCCTCCGAGCAGAACTGAC | | CTGCCTGTGTAGCCATCTGTTGAG |
| *MCSF* | GCTGCCCRTCTTCGACAT | | TCTGACACCTCCTTGGCAATACT |
| *Ctsk* | GGCAGGGTCCCAGACTCCATC | | TGAAAGCCCAACAGGAACCACAC |
| *Nfatc1* | GAGACAGACATCGGGAGGAAGA | | GTGGGATGTGAACTCGGAAGA |
| *Vgf* | ATGACGATGAGGATGCCCAC | | GGCAACGTGAAGGTTTTCAT |
| *Trap* | GGCAGGGTCCCAGACTCCATC | | TGAAAGCCCAACAGGAACCACAC |

**Table S3** Sequences of Primers used for RT-qPCR (human)

| Primer | Forward | **Reverse** |
| --- | --- | --- |
| *GAPDH* | ACATCAAGAAGGTGGTGAAGCA | CGTCAAAGGTGGAGGAGTGG |
| *ANGPT1* | ACTAGTAGTACAATGACAGTTTTCCTTTCC | AGATCTTCAAAAGTCCAAGGGCCGGATCAT |
| *VEGFA* | CCATCCAATCGAGACCCTGG | GGCACACAGGATGGCTTGAA |
| *HIF1A* | CCATTAGAAAGCAGTTCCGCAAGC | GTGGTAGTGGCATTAGCAGTAG |
| *NOS3* | TCACCTTCTTCCTGGACATCACTC | ACCACTTCCACTCCTCGTAGCG |
| *CDH1* | CGCTGCTGCTGCTGCTGCT | TGCTGCTGCTGCTGCTGCT |
| *NCAM1* | GTACCGAGCGCTCTCCTC | ATGACGATGAGGATGCCCAC |
| *PAX6* | AGTGAATCAGCTCGGTGGT | ATTCGGGAAATGTCGCACG |
| *TUBB3* | CAACGCCACGCTGTCCATCC | GCGGAAGCAGATGTCGTAGAGC |
| *RBFOX3* | ATGGCCCAGCCCTACCCC | TCACATGGTTCCAATGCTGT |

**Table S4** Sequences of siRNA

| Name | Forward | Reverse |
| --- | --- | --- |
| mVgf-1992 | AGGAGGAGCUGGAGAAUUATT | UAAUUCUCCAGCUCCUCCUTT |
| mVgf-1617 | UCGAAGAGGUGGAGGAGAATT | UUCUCCUCCACCUCUUCGATT |
| mVgf-1788 | GGGAGGAGGAUGAGGUGUUTT | AACACCUCAUCCUCCUCCCTT |
